# 9S1R nullomer peptide induces mitochondrial pathology, metabolic suppression, and enhanced immune cell infiltration, in triple-negative breast cancer mouse model

**DOI:** 10.1016/j.biopha.2023.115997

**Published:** 2023-12-20

**Authors:** Nilufar Ali, Cody Wolf, Swarna Kanchan, Shivakumar R. Veerabhadraiah, Laura Bond, Matthew W. Turner, Cheryl L. Jorcyk, Greg Hampikian

**Affiliations:** a Department of Biological Sciences, Boise State University, Boise, ID, USA; b Department of Orthopaedics, University of Utah, Salt Lake City, UT, USA; c Biomolecular Research Center, Boise State University, Boise, ID, USA; d Center of Biomedical Research Excellence in Matrix Biology, Boise State University, Boise, ID, USA; e Biomolecular Sciences Graduate Programs, Boise State University, Boise, ID, USA; f Department of Biomedical Sciences, Jaon C. Edwards School of Medicine, Marshall University, Huntington, WV, USA

**Keywords:** Mitochondria, Breast cancer, Triple negative breast cancer, TNBC, Nullomer, Peptide, Tumor microenvironment, Immune cell infiltration

## Abstract

Nullomers are the shortest strings of absent amino acid (aa) sequences in a species or group of species. Primes are those nullomers that have not been detected in the genome of any species. 9S1R is a 5-aa peptide prime sequence attached to 5-arginine aa, used to treat triple negative breast cancer (TNBC) in an in vivo mouse model. This unique peptide, administered with a trehalose carrier (9S1R-NulloPT), offers enhanced solubility and exhibits distinct anti-cancer effects against TNBC. In our study, we investigated the effect of 9S1R-NulloPT on tumor growth, metabolism, metastatic burden, tumor immune-microenvironment (TME), and transcriptome of aggressive mouse TNBC tumors. Notably, treated mice had smaller tumors in the initial phase of the treatment, as compared to untreated control, and diminished in vivo and ex vivo bioluminescence at later-stages - indicative of metabolically quiescent, dying tumors. The treatment also caused changes in TME with increased infiltration of immune cells and altered tumor transcriptome, with 365 upregulated genes and 710 downregulated genes. Consistent with in vitro data, downregulated genes were enriched in cellular metabolic processes (179), specifically mitochondrial TCA cycle/oxidative phosphorylation (44), and translation machinery/ribosome biogenesis (45). The upregulated genes were associated with the developmental (13), ECM organization (12) and focal adhesion pathways (7). In conclusion, our study demonstrates that 9S1R-NulloPT effectively reduced tumor growth during its initial phase, altering the TME and tumor transcriptome. The treatment induced mitochondrial pathology which led to a metabolic deceleration in tumors, aligning with in vitro observations.

## 1. Background

The chance of a woman being diagnosed with breast cancer (BC) during her lifetime is 1 in 8 [1]. In the US, invasive breast cancer is predicted to be diagnosed in an estimated 297,790 women and 2800 men in 2023 [2] and by 2040, with no major changes in prevention or treatment, 1.4 million women will die from BC worldwide [3]. There is a well-established heterogeneity in BC subtypes with presence, absence or a combination of estrogen receptor alpha (ERɑ), human epidermal growth factor receptor (HER2) and progesterone receptors (PR), that dictate treatment strategies. Based on SEER (Surveillance, Epidemiology, and End Results) [4] data in the United States 12% of breast tumors are triple-negative, which lack ER-, PR- and HER2- (triple-negative BC -TNBC) [5]. This subtype is the most aggressive, with the highest level of invasiveness and worst prognosis, resulting in 25–46% of brain metastasis [4]. BC treatment consists principally of surgery, radiation therapy, chemotherapy, hormonal therapy, targeted antibody or small-molecule therapy. Ground breaking approaches such as CAR-T cell therapy show only modest effects on solid tumors [6] and few drugs have been approved by the U.S. FDA for patients with TNBC tumors. PD-1 (programmed cell death protein-1) inhibitor in combination with chemotherapy is one such drug but is limited to patients with tumors expressing PD-L1 (programmed cell death-ligand 1), and those with high-risk, early stage TNBC [7]. The only approved targeted therapy for TNBC is a Trop-2 targeted antibody-and topoisomerase I inhibitor conjugate, used as a second-line treatment for unresectable locally advanced or metastatic triple-negative breast cancer (mTNBC) [8,9]. Recent cancer immunotherapy drug development is focused on immunological agents that augment the natural immune response of patients against cancer-specific antigens [10]. While therapies involving adjuvant immunotherapies, oncolytic viruses, cytokines, antibodies, peptides, and their combinations are in clinical trials for BC treatment, there are certain drawbacks associated with each such as limited efficacy, lack of response, higher cost and poor accessibility for lower income nations. Peptide-based drugs are showing great promise in clinical studies [11–13]. Peptides offer the advantage of small size, specificity, effects on a broad range of cancers, low toxicity and low manufacturing cost [13]. In the current study, we investigated the therapeutic potential of a novel 10 amino acid nullomer-peptide (NulloP) in a TNBC mouse model.

Nullomers are the shortest absent sequences in a species (or group of species). These can be nucleotide strings, or amino acid strings (Nullopeps, or NulloPs). The set of nullomers absent from the entire biome (as represented in available databases) are called primes (DNA) or peptoprimes (amino acids) [14–16]. Both nullomers and primes are seeds from which other absent sequences can be constructed by adding to either end of the string. The nullomer approach to drug design is based on a simple proposition that the smallest absent sequences can affect reactions when injected into hosts. There are two main hypotheses regarding the effects of injected nullomers on cancer cells, they may be toxic or immune stimulatory. The toxic hypothesis is based on the idea that evolution is the longest running experiment in biology, and selection acts to restrict or amplify the permutations of nucleotide and amino acid sequences that arise by chance. In the most extreme case, sequences that conflict with the basic common elements of cell physiology (ribosomes, mitochondria etc.) may be completely “forbidden” [14,17]. The immunogenic hypothesis is based on the idea that the immune system identifies short sequences in a polymer as self or non-self. We could view the immune system as a nullomer search and destroy mission, thus nullomer-based drugs may increase immune responses. The original peptoprime sequences described in 2007 [14] are five amino acids long and have been modified with the addition of five arginine molecules to enhance cell-penetrating properties [17,18]. When dissolved in trehalose, several of the peptoprimes (NulloPTs) were shown to be preferentially lethal to breast and prostate cancer cells, as opposed to normal primary cell lines [17–19]. Trehalose is reported to have an independent effect against BC [20–24] and is an important component in our drug preparation. Nullomers have also been used to design vaccines from epitopes present in the host but absent in a pathogen, and to make DNA “watermarks” for labeling by combining them in series [19].

The original 198 peptoprimes (and scrambled versions of their sequences) were assayed in vitro against the NCI 60 cancer lines [18], and 9S1R, a potent killer of cancer cells was selected for the mouse experiments reported here. 9S1R is a scrambled version of an original peptoprime, that is a nullomer (absent) from the biome. 9S1R-NulloPT is absent from the human and mouse proteomes and has been shown to dramatically decrease ATP production, inhibit mitochondrial F_o_F_1_-ATP synthase, reduce mitochondrial membrane potential and increase superoxide free radicals inside the mitochondria of MDA-MB-231 human TNBC cells and the NCI 60 cancer cell panel, ultimately killing them [17,18].

This is the first in vivo study of nullomer-based peptides. Here we report the therapeutic effects of 9S1R-NulloPT in the 4T1.2-Luc mouse TNBC model. The 9S1R-NulloPT was initially tested in a bilateral tumor model **(pilot study-1, details in** Supplementary) with six administrations at two different doses (50 mg/kg and 100 mg/kg). Following these results, we moved to a unilateral tumor model with eight drug administrations at the most effective dose (100 mg/kg). Although our preclinical in vitro studies showed a substantial response against diverse panels of cancer cells, the present in vivo study in mice showed that the drug effectively reduces TNBC tumor size at the initial growing phase of the tumor, but not in the advanced stage. 9S1R-NulloPT changes the tumor immune microenvironment and affects tumor energy metabolism specifically targeting mitochondrial and ribosomal genes, providing a segue for future studies targeting tumor mitochondria and ribosome synthesis in TNBC.

## 2. Material and methods

### 2.1. Drug preparation-

The 9S1R nullomer peptide (sequence RRRRRWCMNW) was synthesized by Genscript (USA) (lyophilized, HPLC purified, purity > 98%) and stored at − 20 °C. For preparation of 9S1R-NulloPT, briefly, 20 μg/μl stock solution of the 9S1R peptide was prepared in 100 mM Trehalose (Sigma-Aldrich, USA), which was then injected in mice as mg/kg body weight. For example, a 25 g body weight mouse assigned to a dose of 100 mg/kg, received 200 μl of drug formulation containing 100 μg/g bodyweight of 9S1R peptide mixed with 2.5 mM/g bodyweight of trehalose prepared in PBS. Mice in the trehalose only group received 200 μl of 2.5 mM trehalose in PBS per gram bodyweight, and those in the PBS group received 200 μl of PBS. All drug preparations were filtered through a 0.22 μm filter (Millipore, USA) before IP administration.

### 2.2. Single-dose acute toxicity study-

A single dosage of the 9S1R-NulloPT drug at 5, 25, 50, 100 mg/kg and highest equivalent dose of trehalose alone (2.5 mM/g bodyweight) was injected in mice (n = 4) intraperitoneally (IP). Mice were observed every 2 h for clinical signs and symptoms and body weights were taken every 12 h. At the end of the experiment (36 h) mice were euthanized, necropsy was performed and organs preserved in 10% formalin.

### 2.3. Cell culture-

Mouse triple-negative 4T1.2-Luc cells were cultured and maintained as previously described [25] with α-MEM supplemented with 10% fetal clone III serum (Cytiva, USA), 1% penicillin/streptomycin and 1 mM sodium pyruvate. MDA-MB-231 cells were maintained in RPMI 1640 media supplemented with 10% fetal clone III serum (Cytiva, USA) and 1% penicillin/streptomycin (Hyclone, Logan, UT, USA). Cells were maintained at 37 °C, 5% CO_2_, and 100% humidity in a sterile tissue culture incubator.

### 2.4. Generation of the TNBC mouse model and treatment paradigm-

The model was created by orthotopically injecting 4T1.2-Luc cells in the 4th mammary fat pad of Balb/c mice, with 1 × 10^5^ cells per mouse. The cell line was developed by Dr. Cheryl L. Jorcyk [25] and expresses the luciferase gene which serves as a luminescent indicator of gene expression or tumorigenesis, making the tumor cell traceable in vivo. Treatment started after the formation of palpable tumors and was detected by in vivo BLI (IVIS, Perkin Elmer) followed by randomization of the animals per group. For evaluating the effect of the peptides on this model, we carried out two separate studies. The first pilot study involved a set of 4 mice per group with bilateral mammary tumors, which received six IP administration of PBS, 9S1R-NulloPT drug at 50 mg/kg, 100 mg/kg body weight or Trehalose, over a period of 2 weeks. The present study includes a set of 7–9 mice per group with unilateral tumors including the same groups as in the pilot study, but received a total of eight injections per mouse over a period of 2 weeks. The animals were euthanized after 29 days post-tumor cell transplantation, the details are provided in the Supplementary section. The group which received IP injections of 9S1R-NulloPT drug at 100 mg/kg dose is regarded as the treated group, whereas the control group is PBS, unless noted otherwise.

### 2.5. Animal treatment groups-

The 9S1R peptide at dose 100 mg/kg in trehalose is referred to as 9S1R-NulloPT or the treatment group throughout this paper. PBS was used as the control. Results from all groups (PBS, 9S1R-NulloPT at dose 50 mg/kg, 9S1R-NulloPT at 100 mg/kg, and trehalose) are provided in the supplementary materials.

### 2.6. Tumor volume measurement-

Mice were anesthetized by isoflurane followed by measurement of the tumor length and width using a manual Vernier caliper. This was performed before every drug administration and the tumor volume was calculated using the formula (length × width^2^)/2. Body weights were also measured before every dose of the drug.

### 2.7. In vivo bioluminescence imaging (BLI)

Whole-body bioluminescence imaging (BLI) using the In Vivo Imaging System (IVIS^®^ Spectrum, Perkin Elmer) was performed to detect in vivo tumor burden and metastasis, as evaluated by the bioluminescence signal from the 4T1.2-Luc cells. Mice were injected once with 200 μl of D-Luciferin (150 mg/kg) before in vivo BLI. For confirmation ex vivo BLI was performed from the excised tumor, other organs and secondary metastasis sites, when same dose of D-Luciferin was reinjected once more at the endpoint before necropsy and ex vivo BLI. The images were analyzed by Perkin Elmer software and Aura Version 4.0.7 (Spectral instruments Imaging).

### 2.8. Histology of excised tumors-

Half of the tumor tissue was excised and sent for histopathology analysis by a practicing pathologist at the COBRE-Histopathology Imaging Core at the Boise Veterans Affairs Research Department. The tissues were fixed, paraffin-embedded, sectioned (1 μm) and H&E stained. The slides were analyzed for tumor grade, stage, necrosis, aggressiveness, margin inflammation and immune cell infiltration. Representative brightfield microscope images from the H&E stained slides were captured using ECHO Revolve (Bico, USA) and EVOS M50000 (Invitrogen, USA). Images were captured close to the edge of tumor border and stroma of all tumors.

### 2.9. Half-life of peptide 9S1R in serum-

The half-life of peptide 9S1R was determined by spiking the peptide into fetal bovine serum (FBS) for exposure times of 30 s and 5, 30, 60 or 90 min. During the exposure, FBS containing the spike peptides were incubated at 37 °C. The exposure was halted, and the peptide was extracted from FBS using protein precipitation. Protein precipitation was accomplished using 75% ice cold acetonitrile, followed by incubation at − 20 °C for one hour, and centrifugation at 9000 rpm for 10 min at − 4 °C. The supernatant was removed and saved for analysis. Peptide samples were analyzed by High pressure liquid chromatography (HPLC) mass spectrometry (MS) using an ultra-high-resolution Quadrupole Time of Flight (QTOF) instrument (Bruker maxis, Bruker Corporation, Billerica, MA, USA). HPLC mobile phase consisted of 18 MΩ H_2_O and HPLC grade formic acid and acetonitrile (> 99% purity, Fisher Scientific, Pittsburgh, PA, USA). The electrospray ionization (ESI) source was operated under the following conditions: positive ion mode; nebulizer pressure: 1.2 Bar; flow rate of drying gas (N2): 8 L/min; drying gas temperature: 200 °C; voltage between HV capillary and HV end-plate offset: 3000 V to – 500 V; mass range was set from 250 to 2900 *m/z*; and the quadrupole ion energy was 4.0 eV. Low concentration ESI tuning mix (Agilent Technologies, Santa Clara, CA, USA) was used to calibrate the system in the mass range. HPLC separation was achieved using a Dionex UltiMate^®^ 3000 RSLCnano system (Dionex Corporation, Sunnyvale, CA, USA) equipped with a Waters XTerra C18 column (4.6 × 100 mm, 3.5 μm) (Waters Corporation, Milford, MA, USA). The mobile phase was 0.1% formic acid in water (Buffer A) and acetonitrile (Buffer B) with a flow rate of 0.2 ml/min. A linear gradient method was used to separate the mixture starting at 5% acetonitrile and ending at 60% acetonitrile over 20 mins. The sample injection volume was 5 μl. Data were analyzed using the Compass Data Analysis software package (Bruker Corporation, Billerica, MA, USA).

### 2.10. RNA sequencing and transcriptomic analysis from excised tumors-

At termination, tumors (n = 3) were excised, snap-frozen and sent to Novogene for RNA sequencing. Paired-end raw sequences (Illumina platform (PE150) were subjected to quality check using FastQC v0.11.9. Sequence reads for all genes were mapped to the Genome Reference Consortium Mouse build 38 (GRCm38) release 99 by HISAT2 v2.1.0 using Ensemble ID. Aligned sequence reads were assessed by HTSeq v0.11.3 using the GRCm38 (release 99) ensembl reference genome annotation (gtf format) file. Genes with greater than 10 sequence read counts (for each row considering 3 replicates in a sample) per gene were used for further analysis. Differential gene expression for each comparison (3 replicates for each sample) was performed by default Wald test using Deseq2 v1.38.1 in R. Normalization of sequence reads per gene count for each sample was done using median-ratio-normalization. Volcano plots, PCA plots, heatmap of samples (using Euclidean distances), heatmap of expression of top variable genes were generated using gplots, ggplot2 and RColorBrewer programs (R based). Fold-change for each gene was calculated by comparing counts in all the treatments relative to PBS. Genes with Benjamini-Hochberg (BH) adjusted p-value < 0.05 were considered differentially expressed genes (DEGs). DEGs having log2 fold change value of at least 0.58 or greater were used for investigating significant biological processes, molecular functions, cellular compartments and biological pathways using gProfiler web server.

### 2.11. Interactome and cluster analysis from the RNA sequencing data-

To explore if the DEGs are involved in known and predicted protein-protein interactions, the TNBC-related PPI network was constructed using the online analysis tool STRING (Ver 11.2). The network nodes denote the downregulated and upregulated DEGs and network edges shown in confidence view without any disconnected nodes in the network, and with active interaction sources from experiment, databases, co-expression, neighborhood, gene fusion and co-occurrence [26]. The network was clustered to 3 groups following kmeans clustering with hidden edges between clusters for a simplified view. The interaction score was > 0.4 [27].

### 2.12. In vitro Luciferase assay in TNBC cell lines-

4T1.2-Luc cells were plated in white 96 well plates for 48 h without or with 9S1R-NulloPT at concentrations 25, 50 and 100 μM based on previously published IC_50_ ranges [17,18]. At 48 h post treatment, cells were washed in 1 × PBS, exposed to 150 μg/ml D-Luciferin (Thermo-Fisher Scientific, USA) and incubated in PR free media for 10 mins following previously published protocol [25]. Luminescence was measured in a Glomax luminometer (Promega, USA).

### 2.13. Cell viability assay-

Cell viability as a measure of cellular metabolism was examined by MTT assay (Sigma Aldrich, USA) following previously published protocol [28]. Briefly, 4T1.2, 4T1.2-Luc and MDA-MB-231 cells were cultured in 96 well plates for 48 h without or with 9S1R-NulloPT at concentrations 25, 50 and 100 μM based on previously published IC_50_ ranges [17,18]. Post treatment cells were exposed to MTT (Invitrogen, USA) at 5 mg/ml concentration in cell culture media and incubated for 3 h at 37 °C, 5% CO_2_, and 100% humidity. Following media removal, the purple formazan crystals were solubilized in 100 μl DMSO per well and absorbance was taken at 560 nm using a microplate reader (BioTek, Agilent, USA).

### 2.14. In vitro mitochondrial physiology assay-

Measurement of mitochondrial reactive oxygen species (ROS) production was performed using MitoSox dye (Invitrogen, USA), and mitochondrial membrane potential was measured by proton gradient specific TMRM dye (Invitrogen, USA), following previously published protocol [26,29]. Briefly, cells were grown in opaque 96 well plates and incubated with 2.5 nM MitoSox for 15 mins, or 20 nM TMRM for 30 mins at 37 °C, 5% CO_2_, and 100% humidity. Fluorescence was measured using a microplate reader (BioTek, Agilent, USA) at an excitation of 488 nm and emission of 510 nm for MitoSox and at an excitation of 540 nm and emission of 570 nm for TMRM.

### 2.15. Confocal imaging of TAMRA tagged 9S1R NulloPT uptake in vitro-

9S1R tagged with Tetramethylrhodamine (TAMRA) at its N-terminus was procured from Genscript (USA) lyophilized, HPLC purified and with > 98% purity. MDA-MB-231 and 4T1.2-Luc cells grown overnight at confocal dishes were treated with nontoxic dose (20 μM) of TAMRA-9S1R NulloPT or trehalose control, following previously published short poly arginine peptide uptake protocol [30,31]. The cells with or without peptide were incubated at 37 °C for 2 h in serum free media, followed by washing in PBS. Hoechst 33342 (Invitrogen, USA) at 1x concentration was added to the cells and incubated for 15 min for counterstaining the nuclei. After washing with PBS, live cell images were captured in PR free complete media (GIBCO, USA) using a Leica Stellaris 5 Confocal microscope with attached Uno stage top incubator, which maintains cells at 37 °C and 5% CO_2_. Z-stack images were captured at 40 × magnification using a water immersion objective and maximum intensity projections were created using Fiji software (Ver 1.54 f).

### 2.16. Statistical analysis-

Two-tailed unpaired Student’s t-test was used for experimental statistics with p < 0.05 [31]GraphPad Prism (ver 9.5.1) was used for analysis.

## 3. Results

### 3.1. Characterization of the peptide 9S1R

**Fig. 1.**
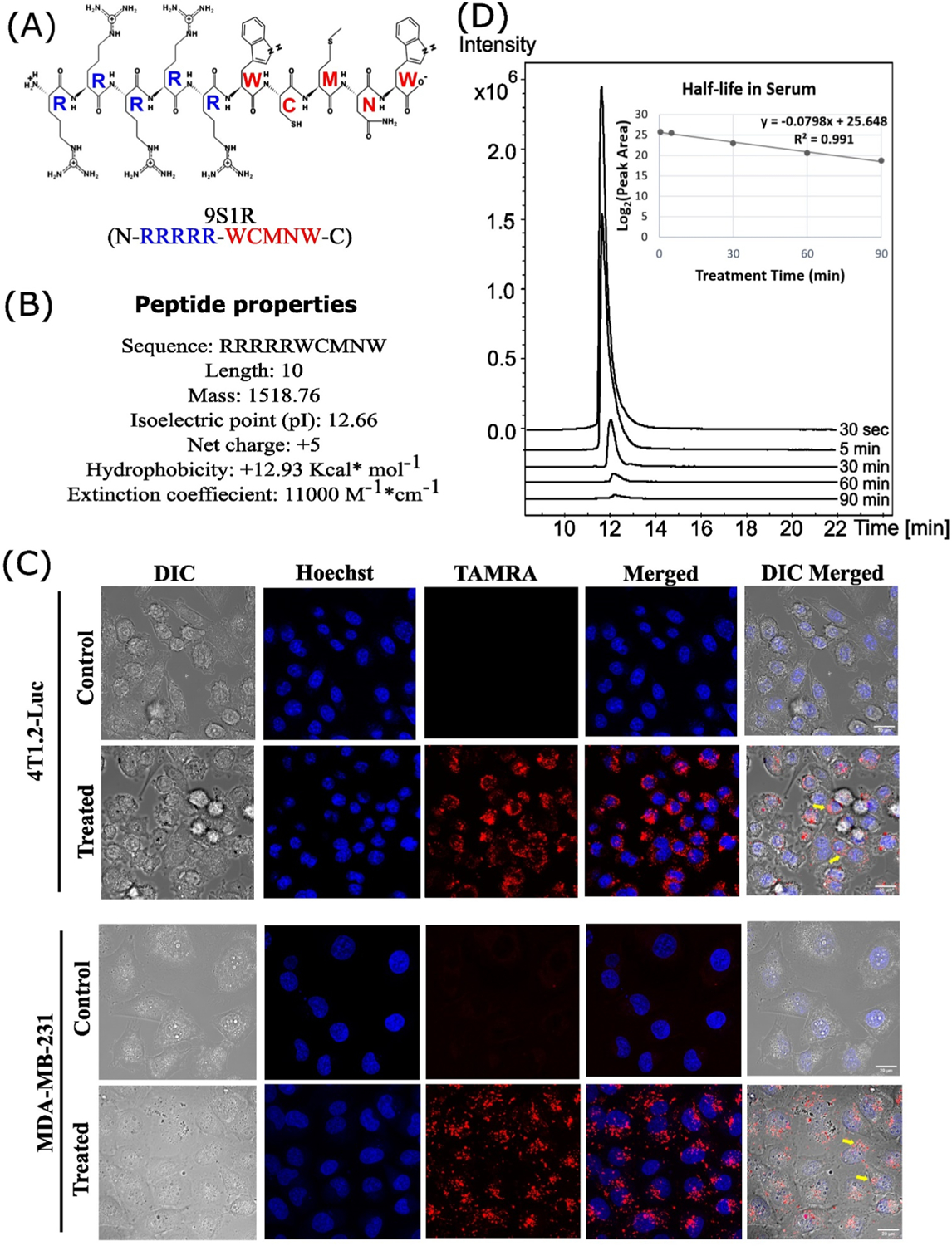
Characterization of 9S1R nullomer peptide. (A) Projected biochemical structure, blue represents poly-arginine and red is the 5 aa nullomer sequence (WCMNW). (B) physical properties of the peptide as obtained from Pepdraw (©2015). (C) Maximum intensity projection images of confocal sections from 4T1.2-Luc and MDA-MB-231 cells treated with non-toxic dose of 20 μM TAMRA labeled 9S1R NulloPT (red) or unlabeled trehalose control. Nuclei stained with Hoechst 33342 (blue). Arrows (yellow) in DIC merged indicate internalized labeled peptide in cells. Images captured at a magnification of 40x, scale bar represents 20 μm. (D) The extracted ion chromatogram (EIC) peaks of the peptide after LC-MS at different incubation times in serum (0.5–90 min). The X-axis depicts the retention time and Y-axis peak intensity. Inset shows the half-life log_2_ peak area under the curve versus treatment time for the EIC peaks. t_1/2_ value for the peptide is 8.7 min.

The nullomer 9S1R (N-RRRRR-WCMNW-C) has a molecular weight of 1519.81 Da and iso-electric point of pH 12.28. It has a net charge of + 5 at pH 7, hydrophobicity + 12.93 Kcal/mol with amphipathic nature and fair solubility in water (Fig. 1A, B). We evaluated the internalization and cellular uptake of TAMRA labeled 9S1R NulloPT after 2 h of incubation in 4T1.2-Luc and MDA-MB-231 cells by confocal microscopy (Fig. 1 C). We found at physiological condition and at a non-toxic dose (20 μM) [30,31] the peptide appears to be internalized within the cells, through endocytosis and primarily located at the cytosol, which is typical for short polyarginine containing peptides [32–34]. We further confirmed the uptake of the native untagged peptide inside 4T1.2 cells by mass spectrometry (MS) and found that the peptide is available inside the cells at two molecular forms with charges 3 + (*m/z* 507.2637 Da) and 4 + (*m/z* 380.7002 Da), (extracted ion chromatogram (EIC) peaks, Supplementary Fig 1). We determined using MS that the half-life of the peptide in fetal bovine serum (FBS) was 8.7 mins (Fig. 1D). The previous in vitro study by Alileche et al. showed that this peptide targets mitochondrial function [18]. This agrees with the above properties of this molecule, in that it is very similar to known mitochondria-targeting peptides (MTPs) [35–39] and, we hypothesize that the peptide ultimately localizes to mitochondria after its endosomal processing in the cytosol. Thus, we determined that the peptide enters cells and has a short half-life in serum.

### 3.2. Single dose acute toxicity study of 9S1R NulloPT in mice

**Fig. 2.**
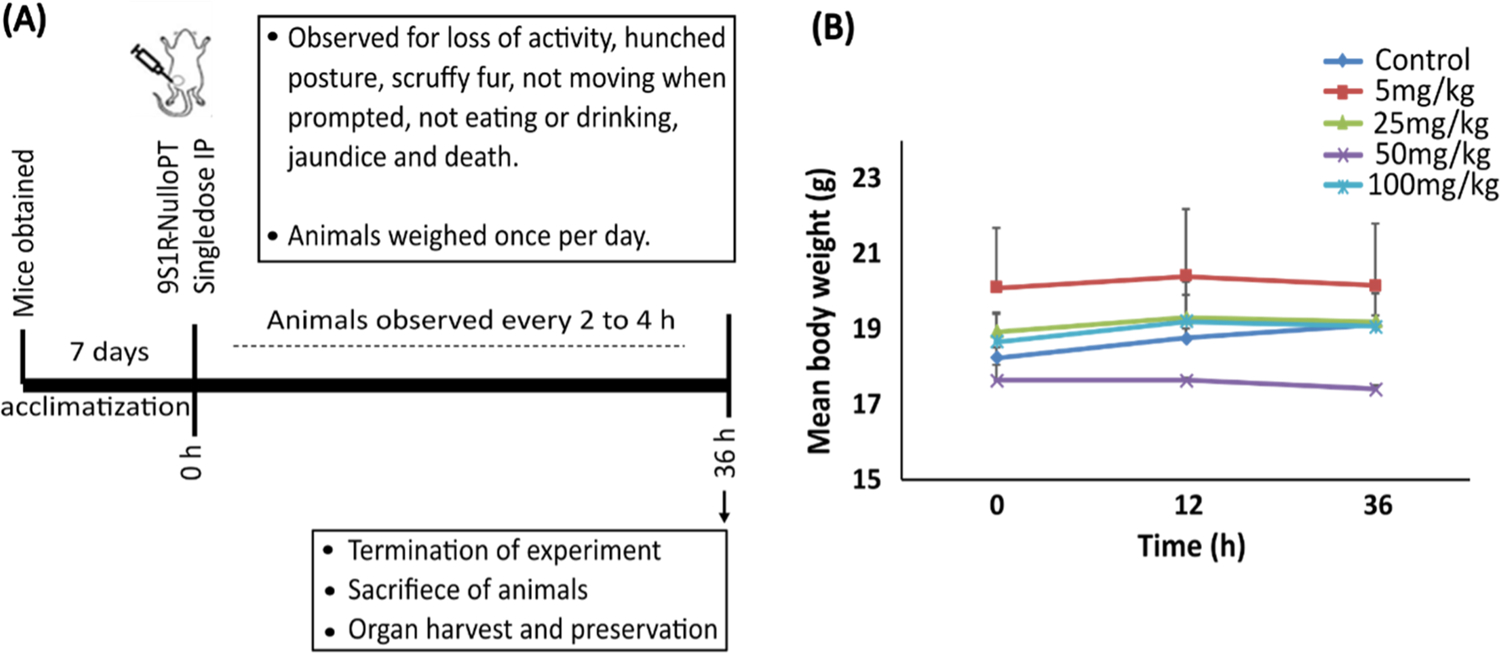
Toxicity study in mice (A) In vivo safety study timeline. (B) Changes in mean body weight plotted against time among mice treated with trehalose alone or 9S1R-NulloPT drug at 5, 25, 50 and 100 mg/kg; N = 3.

To determine the safety of our peptide formulation in vivo, we performed a single dose toxicity study in female BALB/c mice (Fig. 2 A). The drug doses (administered I.P.) used are 5, 25, 50 and 100 mg/kg, with the control being the highest trehalose dose volume. After 36 h of observation the experiment was terminated, and we found no significant change in the animal behavior, clinically observable signs of acute toxicity or conspicuous change in body weights among any groups (Fig. 2B). There was no significant difference between the drug and trehalose alone group. All the drug doses were well tolerated by the animals, allowing us to move forward with the preclinical in vivo cancer studies.

### 3.3. Treatment with 9S1R-NulloPT inhibits tumor growth in early stage cancer but does not affect tumor volume in late stage growth

**Fig. 3.**
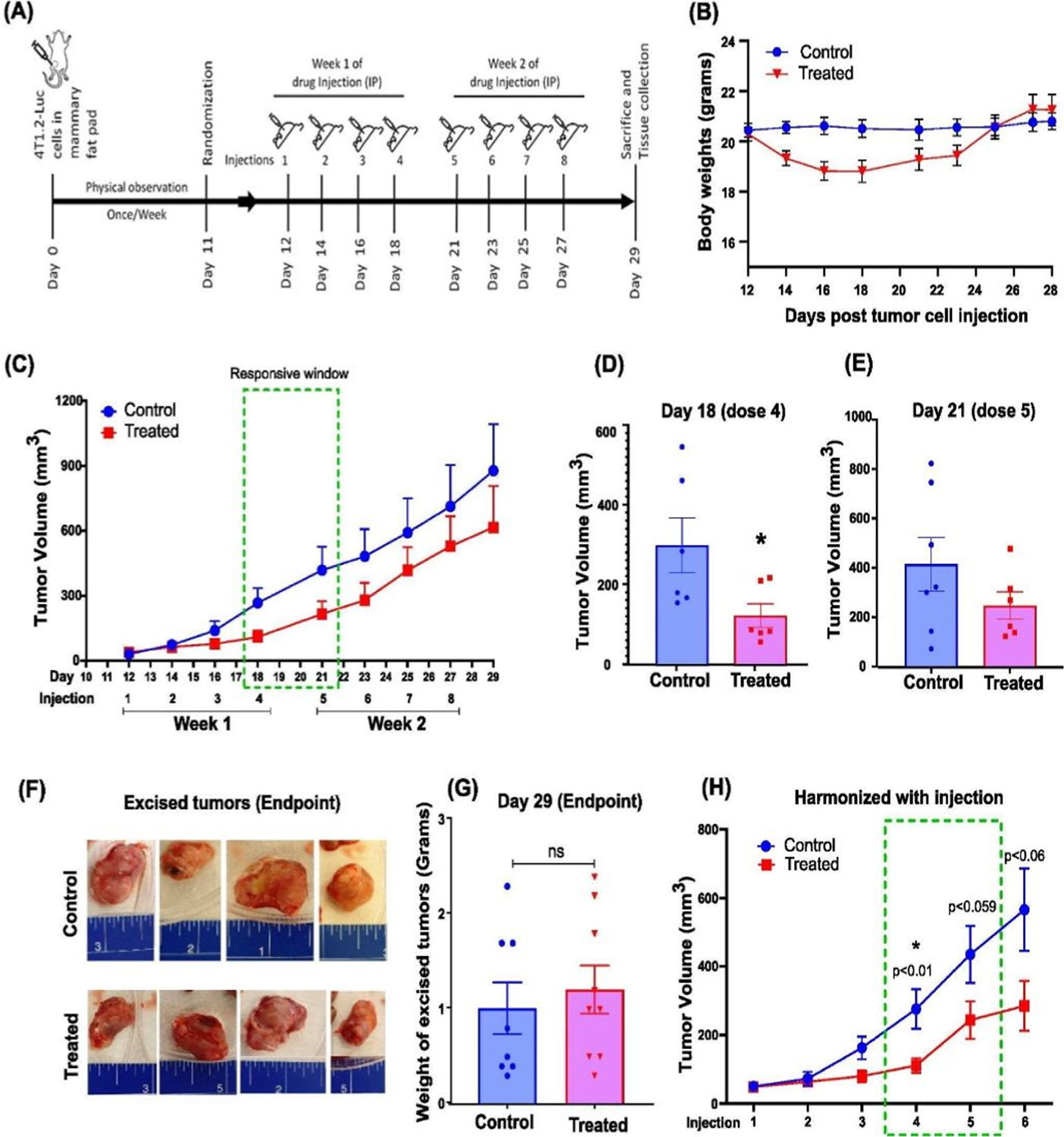
Effect of 9S1R-NulloPT on tumor volume: (A) Treatment schedule. (B) Body weight over time, in control and 9S1R-NulloPT treated mice. (C) Tumor volume by caliper measurement plotted against days post tumor cell injection with injection timeline of 8 doses, green box highlights drug-responsive therapeutic window with highest inter-group difference. (D) Cross-sectional analysis by Tumor growth [40] showing the highest responsive window on day 18, 4th dose (p < 0.03, n = 6–7) and (E) day 21, 5th dose (p < 0.2, n = 7). (F) Excised tumors at endpoint from control and treatment groups; blue scale in inches. (G) Change in excised tumor weight from control and treatment groups. (H) Tumor volume measurement by harmonizing the data from both current and pilot studies, data aligned by injection timepoints. The green box depicts the responsive window with a statistically significant difference between the control and treated groups by the 4th dose (p < 0.01, n = 10–11). Data presented as Mean+ SEM followed by two-tailed unpaired Student’s t-test, *p < 0.05 considered statistically significant.

We evaluated the effect of 9S1R-NulloPT on female BALB/c mice (8 weeks old), using a syngeneic 4T1.2-Luc orthotropic model of metastatic TNBC. We investigated the effect of the drug on tumor growth over one month in both a bilateral model (initial pilot study, Supplementary Fig 2.) and a unilateral model (Fig. 3A and Supplementary Fig 3). In both studies, there was a decrease in weight following the first week’s treatment, however the mice later returned to near-control level weight (Fig. 3B, Supplementary Fig 2 C). The weight loss was never greater than 15% of the initial body weight, ruling out any adverse effects. We found that although the 9S1R-NulloPT treated mice maintained a reduced tumor volume as compared to untreated controls (Fig. 3C, Supplementary Fig 2B), the decrease was statistically significant only at the fourth dose (day 18), with a highest inter-group difference (evaluated by Tumgrowth [40]) of 2.4 fold (p < 0.03), followed by the fifth dosage (day 21) with a difference of 1.7 fold (p < 0.2, not significant) (Fig. 3 D, E). There was no statistically significant decrease in any other timepoints, with inter group difference at the third dosage (day 16) being 1.5-fold (p < 0.3), sixth dosage (day 23) 1.7-fold (p < 0.2), seventh dosage (day 25) 1.4-fold (p < 0.3) and eighth dosage (day 25) 1.4 fold (p < 0.4). This indicates a 9S1R-NulloPT therapy responsive window between doses 4 and 5 (Day 18–21) [40]. At the endpoint on Day 29, the size and weight of the excised tumors showed no significant difference between the treated and control groups (Fig. 3F, G, Supplementary Fig 3A–D). After harmonizing (aligning the injection days), the combined results of the two studies show a significant decrease in tumor size for the treatment group, with a difference of 2.5 fold (p < 0.015, dose 4), 1.8 fold (p < 0.06, dose 5), and ~2 fold (p < 0.06, dose 6) (Fig. 3H) [35, 36,38,39]. This suggests that the 9S1R-NulloPT drug is effective in reducing growth during the early phase of tumor development.

### 3.4. 9S1R-NulloPT alters tumor metabolism but not metastasis

**Fig. 4.**
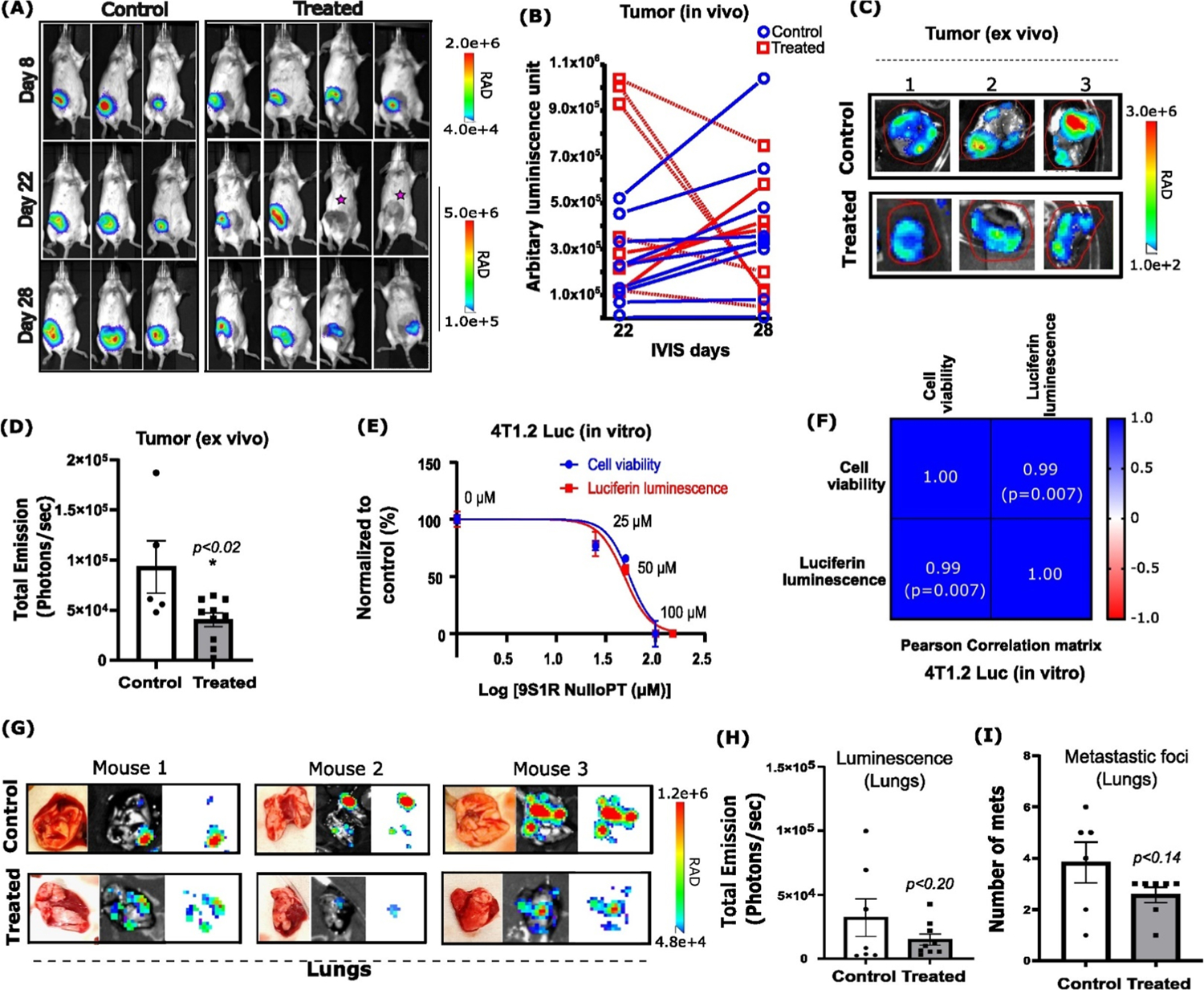
Effect of 9S1R-NulloPT treatment on tumor metabolism and lung metastasis: (A) Representative images taken by IVIS Spectrum (Perkin Elmer) on days 8, 22, and 28 post 4T1.2-Luc cell injection, in control and 9S1R-NulloPT treated mice, showing in vivo bioluminescence signal from the tumor cells. Pink star marks no signal in the treatment group. (B) Bioluminescence intensity derived from in vivo BLI at day 22 and 28 showing treated (red) and control (blue) groups with dotted lines denoting a decrease in intensity from individual mice. (C) Ex vivo tumor images on the same luminescent intensity scale from representative control and treated mice. (D) Tumors from the treated group show statistically significant (p < 0.02) loss of bioluminescence represented as photons/second when compared to control tumors. (E) 4T1.2- Luc cells in culture showing dose dependent decrease in cell viability and luciferin induced luminescence, upon treatment with 9S1R-NulloPT. (F) Pearson correlation matrix showing a positive correlation with r = 0.99 (p = 0.007, N = 4–5) between cell viability and luciferin luminescence as found in E. (G) Representative photographs, images from ex vivo bioluminescent imaging (BLI) and fluorescence only signals from lungs, the secondary metastasis sites of control and treated mice. (H) Intensity analysis of total emission from Lungs BLIs showing a trend of reduction (not-significant, p < 0.20) in bioluminescence after treatment. (I) Total count of metastatic foci in lungs with no significant (p < 0.14) change among the groups. Data presented as Mean+ SEM followed by two-tailed unpaired Student’s t-test, *p < 0.05 considered statistically significant, N = 5–8.

Monitoring bioluminescence of the tumor cells in vivo revealed that, although the initial tumors (day 8) had similar signals in both control and treatment group, the control group tumors displayed increased in vivo bioluminescence with time. Five of 8 treated animals showed a reduction at the later time points (day 22 and day 28) (Fig. 4 A, B). We found that the excised tumor weights among the groups were not significantly different at the end of the study (Fig. 3); however, the ex vivo BLI of the excised tumors (post mortem) showed a significant decrease in signal with treatment (Fig. 4C, D). This suggests that tumors from the treatment group have a decreased number of metabolically active 4T1.2-Luc cells. Firefly luciferase oxidizes luciferin by using ATP, Mg^2 +^ and O_2_ [41], and depletion of cellular ATP results in lower luminescence along with loss of metabolic functioning. It has been previously shown in vitro that 9S1R-NulloPT completely depletes the cellular ATP of BC cell lines within 3 h and are highly lethal to hormone independent and triple negative BC cell lines (MDA-MB-231, BT-549, HS-578 T, and MDA-MB-468), as well as hormone dependent BC cell lines (MCF-7 and T-47D) [17,18]. The direct association between decrease of luminescence with changes in cell viability was confirmed in cultured 4T1.2-Luc cells (Fig. 4 E, F). We observed that the 9S1R-NulloPT induced a dose-dependent loss of luminescence and a decrease in cell viability as measured by cellular metabolic activity using MTT assay [42] (Fig. 4E). Additionally, these decreases were found to be significantly positively correlated with each other (Fig. 4 F).

Although there was a trend of reduction, there was no significant change in the bioluminescence of cells metastasizing to lung (Fig. 4 G, H) and no change was seen in the number of metastasis to lungs after treatment (Fig. 4 I). These findings corroborated our previous study (Supplementary Fig 2 A–I), and support the role of 9S1R-NulloPT in altering tumor metabolism yet having no conclusive effect on metastasis [35,36,38,39].

### 3.5. 9S1R NulloPT alters tumor immune microenvironment

**Fig. 5.**
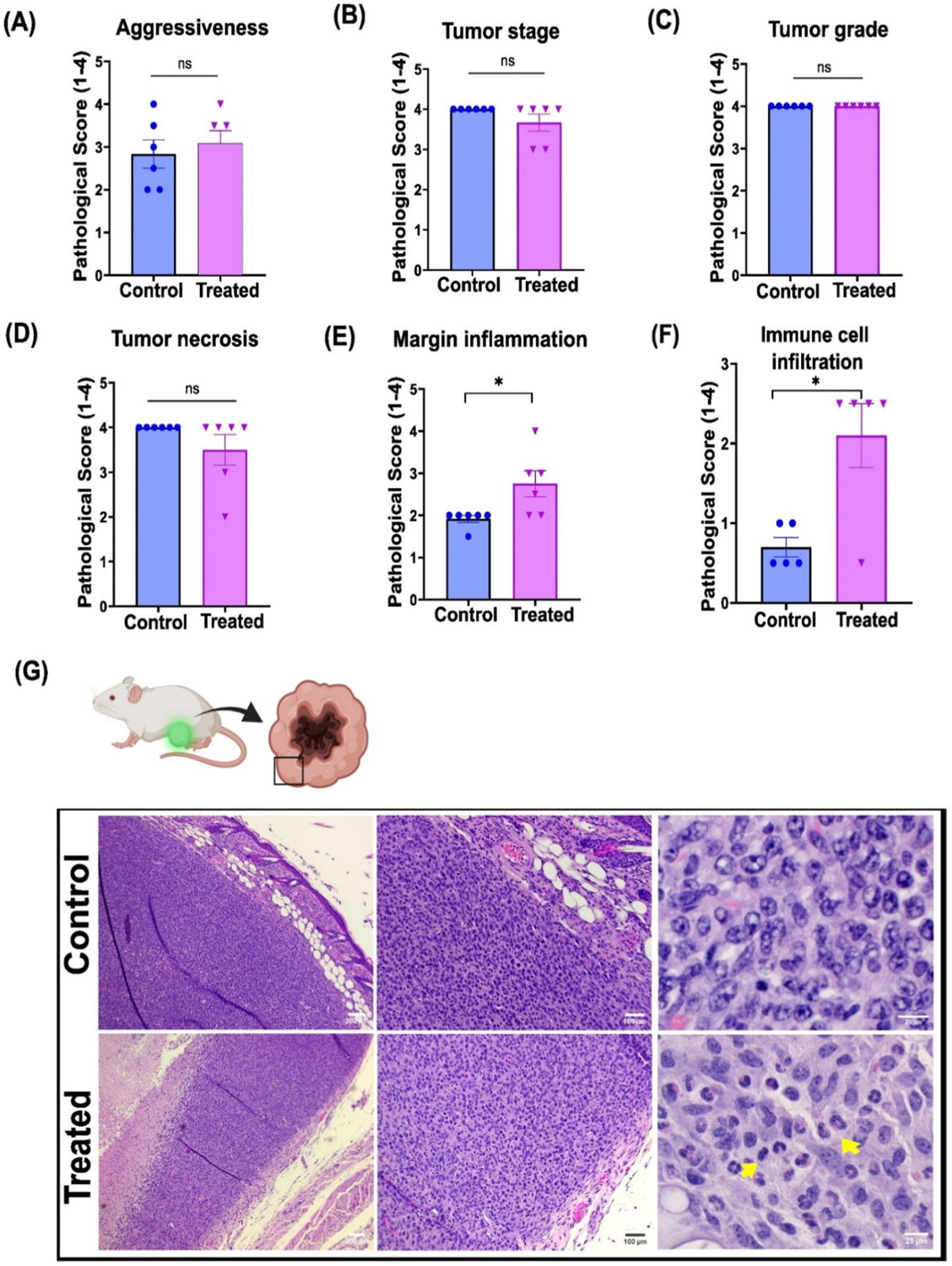
Tumor histology: Pathological scoring from the Hematoxylin and Eosin stained sections of mammary tumors from control and treated mice, revealing (A) aggressiveness of the tumor, (B) stage of tumor, (C) tumor grade, (D) score of observed necrosis, (E) marginal inflammation of tumor, and (F) infiltration of immune cells within the tumor. (G) Representative image from the sections at 4x (scale 200 μm), 10x (scale 100 μm) and 40x (scale 25 μm) magnification, showing increased immune cell infiltration in the treated tumor (yellow arrows). Data represented as Mean ± SEM, Two-tailed student’s t test with * P ≤ 0.05, N = 5–6.

Histopathological scoring of tumor grade and stage (on a scale of I-IV) from the H&E stained sections was performed (similar to that used for human breast tumors), per NCI’s recommendation. All the 4T1.2-Luc tumors were of very advanced stage, and had a necrotic center. Although tumors from all groups were similar in aggressiveness via grade and stage (Fig. 5 A–D), there was ~1.5-fold increase in margin inflammation, with presence of inflammatory cells on tumor margins in the treated group. There was a 3-fold increase in the immune cell infiltration within the treated tumors compared to the control untreated group (Fig. 5E–G). The pathological report also suggests infiltration of plasma cells in the treated tumors, while none were found in the control groups. The results indicate that 9S1R-NulloPT treatment alters the TME and enhances the immune and inflammatory response. This is corroborated by the increased number of plasma cells [43], tumor-infiltrating lymphocytes (TILs) [44,45] and higher immune scoring [46], which are all associated with a positive prognosis in TNBC patients.

### 3.6. RNA seq, transcriptomics and network analysis of treated TNBC tumors

**Fig. 6.**
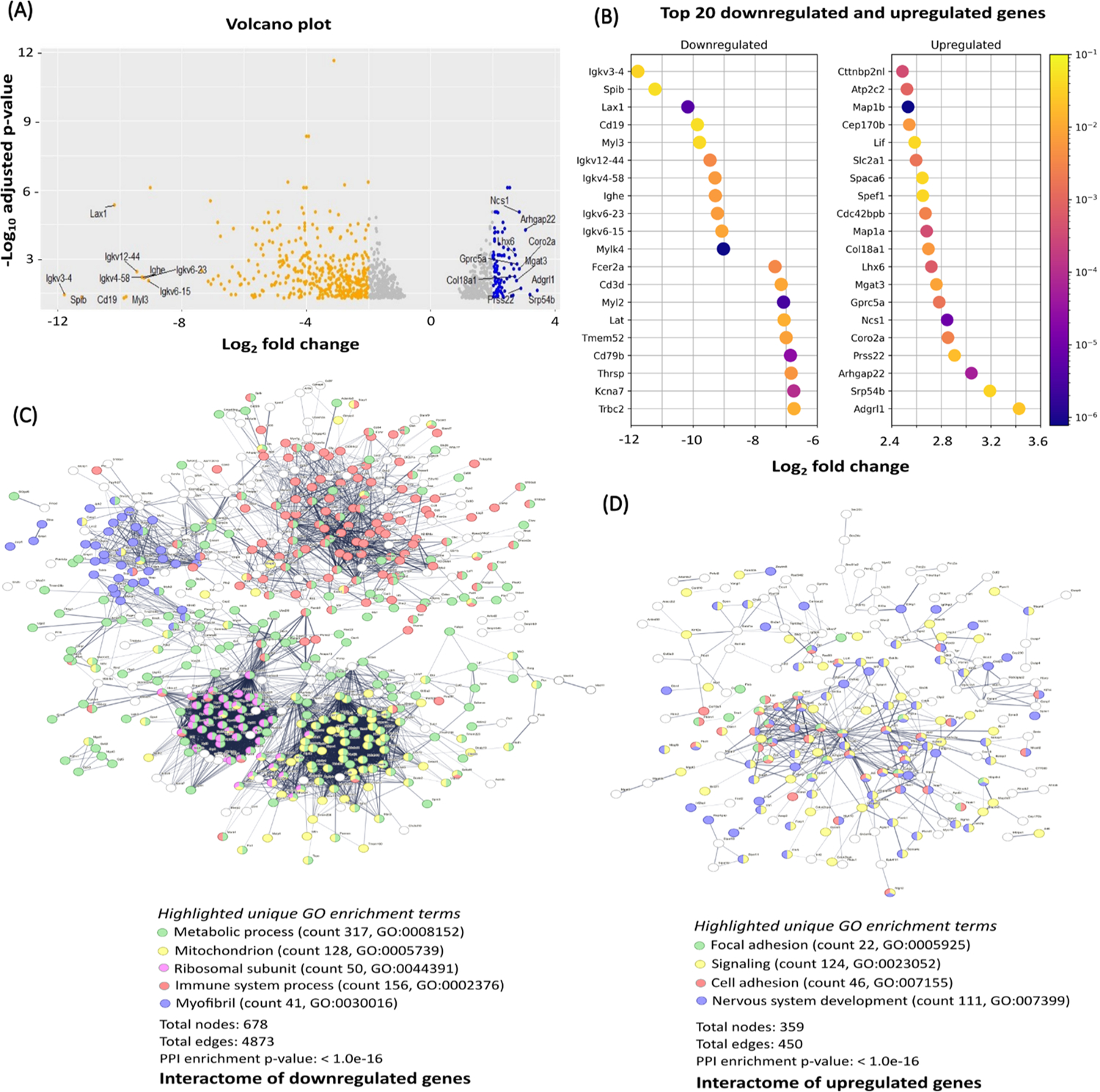
RNA sequencing and network analysis: RNA sequencing from advanced stage TNBC tumors (n = 3) showing the effect of 9S1R-NulloPT treatment. (A) Volcano plot showing total DEGs with top 10 upregulated (orange) and downregulated (blue) genes. (B) Top 20 downregulated and upregulated DEGs and their Log2 fold change values (X-axis) and the p-adjusted values (padj) represented as heat map. (C-D) Interactome of the genes following STRING analysis showing DEGs involved in known and predicted protein-protein interactions with network nodes representing upregulated (C) and downregulated genes (D). Network edges (protein-protein interactions) shown in confidence view, the unique enriched GO terms are highlighted to best represent the clusters with counts and GO IDs for upregulated and downregulated interactome.

To better understand the effects of 9S1R-NulloPT treatment in advanced stage tumors, we performed post-mortem RNA sequencing followed by transcriptomics analysis. The overall gene downregulation was much higher than the level of upregulation, upon treatment, 365 genes were upregulated and 710 genes were downregulated. The top five upregulated genes (with respective Log2 fold change value) were Adgrl (3.4), Srp54b (3.2), Arghgap22 (3.0), Prss22 (2.9) and Coro2a (2.8); and the top five downregulated genes were Igkv 3–4 (−11.8), Spib (−11.2), Lax1 (−10.1), Cd19 (−9.8) and Myl3 (−9.7) (Fig. 6 A–B). The function of these up- and downregulated genes are provided in Supplementary Table 1.

Interestingly the Igkv (immunoglobulin kappa variable) family (Igkv 12–44, Igkv 3–4, Igkv 4–58, Igkv 6–15, Igkv 6–23 and Ighe) were among the top 10 downregulated genes (Fig. 6A). We investigated the presence of clusters in the interactome network of the differentially expressed genes (DEGs) from total upregulated and downregulated genes, using STRING software (Ver 11.5) [27]. Of the upregulated DEGs, we found clusters specific to cancer, focal adhesion, signal transduction, cell adhesion and nervous system development (Fig. 6C). Network analysis of downregulated genes revealed an overall cluster for metabolic process related genes with specific clusters for mitochondria, ribosome and translation machinery, immune system and myofibril assembly (Fig. 6D). Functional enrichment analysis using the five highest significant gene ontology (GO)-terms (biological process, molecular function and cellular component) and pathways (from Kegg, Reactome and Wikipathways) of upregulated and downregulated DEGs confirmed the results [47,48]. The details of the enrichment analysis and list of pathways are provided in Table 1.

### 3.7. 9S1R-NulloPT specifically targets metabolic and bioenergetics pathways

**Fig. 7.**
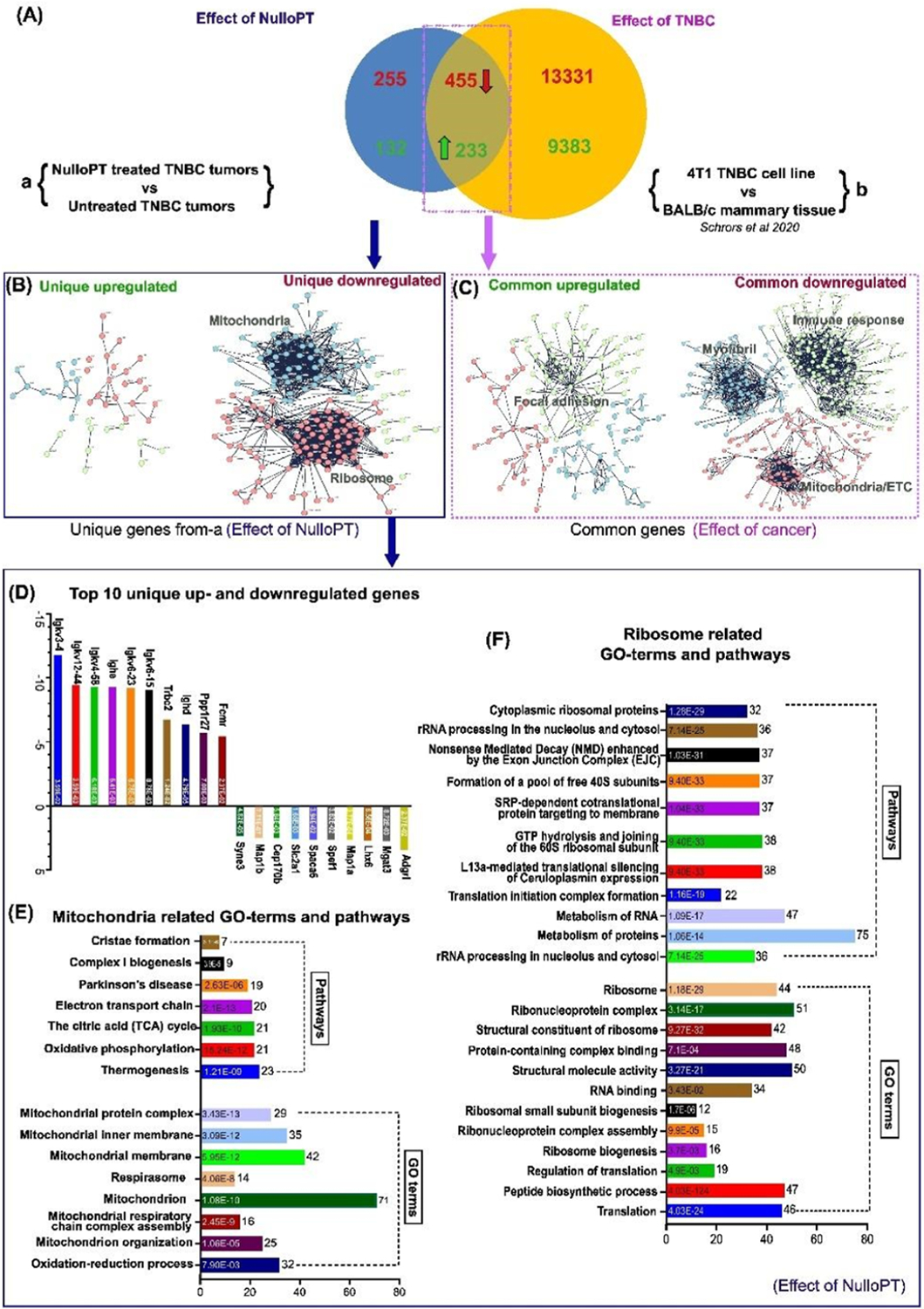
Comparative analysis of the effect of cancer vs effect of 9S1R-NulloPT treatment, in mice mammary tissue: DEGs from a published study [49] comparing 4T1.2-Luc TNBC cells originating from BALB/c mammary gland vs normal BALB/c mammary tissue (group-b). These were compared with the DEGs obtained from the present study of 4T1.2-Luc tumors treated with 9S1R-NulloPT vs untreated tumors (group-a) to differentiate the drug-induced changes vs cancer-induced changes on the mammary tissue. (A) 132 upregulated and 255 downregulated genes were unique to group-a as an effect of 9S1R-NulloPT treatment, 455 genes were commonly downregulated and 233 commonly upregulated in the two groups, and thus attributed to cancer-related effects. (B) Cluster analysis of the unique DEGs from our study (group a) emphasizes the drug-induced changes. No definite cluster of upregulated genes were found; however, the downregulated genes show two prominent clusters of Mitochondria related genes and Ribosome assembly/Translational machinery related genes. (C) Results from cluster analysis of the common DEGs emphasizing cancer induced gene expression, with focal adhesion related cluster upregulated and at least three distinct downregulated clusters comprising Mitochondrial Electron Transport Chain genes, Immune system-related genes, and Myofibril assembly genes. (D) Analysis of unique clusters from group-a (effect of 9S1R-NulloPT) revealed a list of top 10 up- and downregulated genes with Log2 fold change values (Y-axis) and padj scores inside the columns; a list of unique GO-terms (including biological processes, molecular function, cellular components) and pathways (including Kegg, Reactome and Wikipathways) with number of genes (X-axis) and FDR values inside columns for (E) Mitochondrial cluster and (F) Ribosomal cluster.

We found cancer related pathways, and at least 65 cancer pathway-associated DEGs, were upregulated with treatment. Since, we compared treated TNBC tumors with untreated TNBC tumors, we investigated whether such cancer related pathways and DEGs are contributions of the cancer model or an effect of drug-induced change. To achieve this, we used publicly available RNA-Seq data by Schrors et al. [49], comparing gene expression of 4T1.2-Luc TNBC cells (derived from BALB/c mammary gland) versus normal BAlb/C mammary tissue. This comparison identifies the DEGs that result from BC (TNBC) alone. Our 4T1.2-Luc cell line, developed by Jorcyk et al. [25], is a luciferase tagged single cell clone of 4T1.2 cells [50] with similar genomic background and tumor properties to the original 4T1, but with a higher tendency to metastasize. A comparison of the DEGs from 9S1R-NulloPT treated vs untreated tumors (group a), and 4T1 tumor cells vs mammary cells (group b) identified those DEGs that resulted from 9S1R-NulloPT treatment (unique from group a). DEGs resulting from TNBC [51,52] are common to group a and b (Fig. 7).

The comparison between the RNA-Seq results revealed that 688 DEGs were due to TNBC (common to group a and b), with 233 genes upregulated and 455 genes downregulated (Fig. 7A). Cluster analysis of these genes using STRING (Ver 11.5) revealed the TNBC downregulated clusters consist of myofibril assembly, immune related and mitochondrial electron transport chain (ETC)TC related genes. The TNBC upregulated cluster comprises focal adhesion and cancer signaling related genes (Fig. 7 C). Although no noteworthy cluster was found in upregulated genes unique to 9S1R-NulloPT treated cancer, Abl-1 and Shc-1 formed the center node of a small cluster. Remarkably, when analyzed in details (Supplementary Fig 5), among the above-mentioned cancer related pathways we found only 20 associated genes to be uniquely upregulated in 9S1R-NulloPT treated tumors (Supplementary Fig 5 A–C) including pan-cancer related genes such as Shc-1 [53], Mtor [54], Lrp6 and Wnt5a [55]. Interestingly, a recent report suggests that Wnt5a [56] and Abl-1 [57] are potent suppressors of TNBC progression and are associated with a better prognosis in BC. We also found 44 DEGs associated with at least 12 cancer pathways that were upregulated in both 9S1R-NulloPT treated and TNBC untreated tumors (Supplementary Fig 5, D–F), signifying a contribution from the TNBC model. A list of cancer pathway related DEGs in TNBC untreated tumors and 9S1R-NulloPT treated tumors is provided in Supplementary figure 6.

The unique downregulated clusters, which signify the effect of 9S1R-NulloPT only (and not breast cancer), comprise mitochondrial ATP synthesis coupled proton transport (Atp5e, Atp5j, Atp5k, Atp5h and Atp5j2), mitochondrial electron transport chain (Uqcrb, Uqcrh, Ndufv2 and Ndufs6), Oxidative phosphorylation (Cox6c, Atp5e, Uqcrb, Ndufs6, Atp5j, Cox7a2, Atp5k, Uqcrh, Atp5h, Ndufv2 and Atp5j2), Mitochondrial respiratory chain complex-1(Ndufv2, Ndufs6, Ndufs5, Ndufa5, Ndufb5, Ndufs4 and Ndufb3) and ribosome/translation related genes (Rpl17, Rpl19, Rps27l, Rpl22l1, Rpl9, Rps14, and Rps27) (Fig. 7 B, E, F). The top 5 unique downregulated genes were from the Igkv family (Igkv 3–4, Igkv 12–44, Igkv 4–58, Ighe and Igkv 6–23) and top 5 unique upregulated genes were Adgrl, Mgat3, Lhx6, Map1a and Spef1 (Fig. 7D). Enrichment analysis of the mitochondria cluster revealed Cristae formation, Complex I biogenesis, Oxidative phosphorylation, Electron transport chain, TCA cycle pathways and the GO-terms such as respirasome, mitochondrial respiratory chain assembly and mitochondrion organization (Fig. 7E). Enrichment analysis of the ribosomal cluster revealed Ribonucleoprotein complex, RNA binding, Ribosomal biogenesis, Translation and Peptide biosynthesis pathways and the GO-terms such as rRNA processing, SRP dependent cotranslational protein targeting to membrane, L13a mediated silencing of Ceruloplasmin expression (Fig. 7F). Interestingly, low ceruloplasmin expression correlates with a favorable prognosis and tumor immune cell infiltration in BC patients [58]. We found 149 genes which were upregulated with TNBC (in group-b) were downregulated as an effect of 9S1R-NulloPT treatment (in group-a). These genes comprise the same cluster of Ribosome and Mitochondria genes with similar GO-terms and pathways (Supplementary Fig 6 A–C). Likewise, 99 genes which were downregulated as an effect of TNBC (group-b) were upregulated due to 9S1R-NulloPT treatment (group-a), and these includes Focal adhesion, nervous system development, and ECM organization related genes (Supplementary Fig 6 D–F). In summary, transcriptomic analysis suggests that 9S1R-NulloPT targets metabolic pathway related genes, specifically the Mitochondrial energy metabolism and Ribosome associated pathways such as Ribosome biogenesis and translation.

### 3.8. 9S1R-NulloPT reduces mitochondrial membrane potential and increases mitochondrial ROS in triple negative breast cancer cell lines

**Fig. 8.**
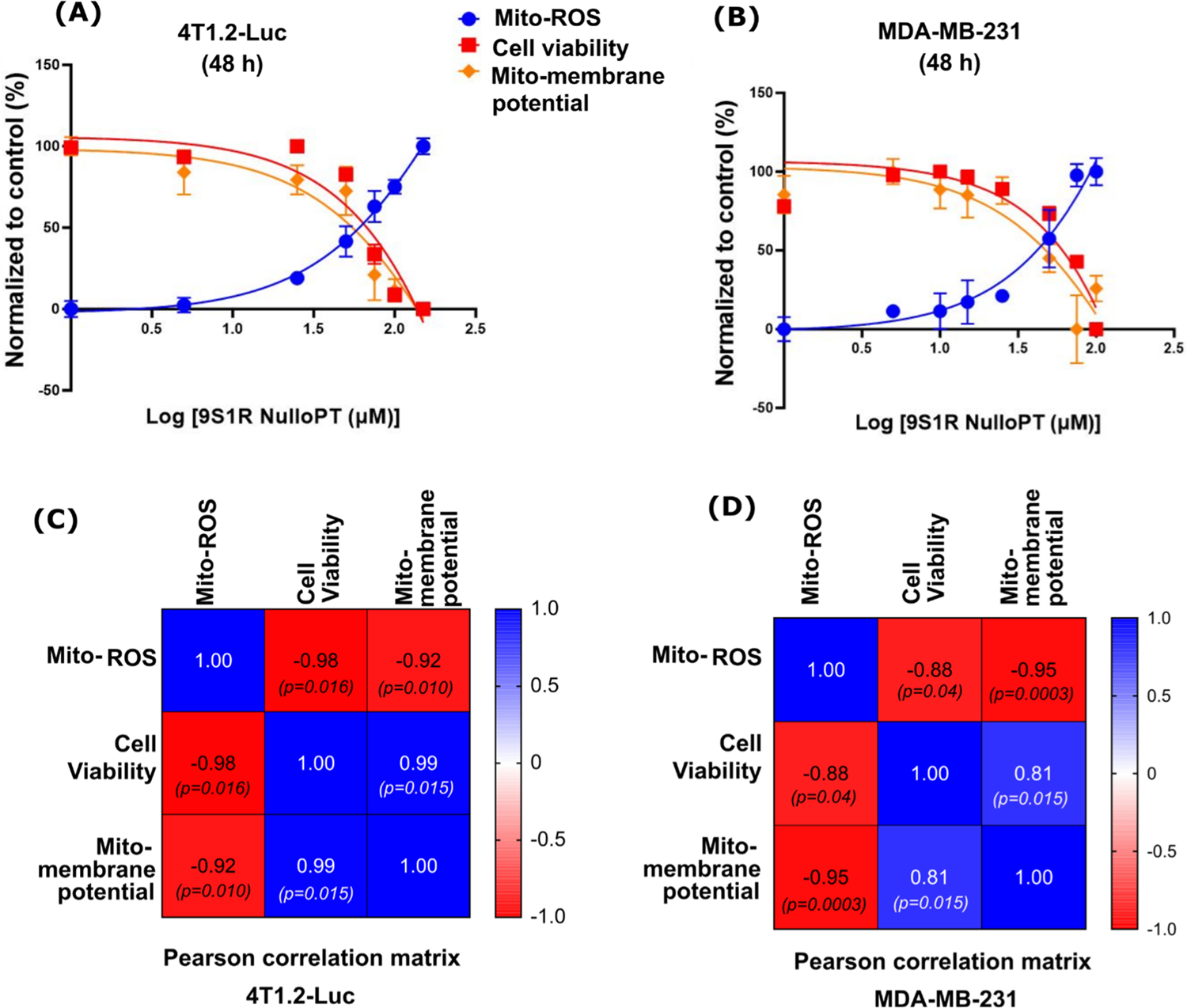
9S1R-NulloPT shows a dose-dependent alteration on mitochondrial physiology and cell viability, in mouse and human TNBC cell lines: 4T1.2-Luc a mouse TNBC cell line (A) and MDA-MB-231 human TNBC cells (B) showing best fit curve indicating a dose dependent reduction in mitochondrial membrane potential, cell viability, and an increase in mitochondrial ROS production, in in vitro cultures. Pearson’s r correlation matrix between mitochondrial membrane potential, cell viability, and mitochondrial ROS, showing positive correlations (blue) and negative correlations (red), with r and p values depicted inside the matrices, in 4T1.2-Luc (C) and MDA-MB-231 (D) cell lines. Heat map for the value of r closer to + 1 (blue) or – 1 (red) shows the degree of positive or negative correlation respectively. Data presented as Mean ± SEM (N = 4–6).

We confirmed the effect of 9S1R-NulloPT on mitochondrial physiology in in vitro cultures of mouse TNBC cell line 4T1.2-Luc and human TNBC cell line MDA-MB-231. We found that in both cell lines (Fig. 8A, B) treatment induces a dose-dependent decrease in cell viability and cellular metabolism as measured by MTT assay [28,42], and a decrease in mitochondrial membrane potential measured by TMRM fluorescence. We also found an increase in the generation of mitochondrial ROS production with an increased dose of 9S1R-NulloPT as indicated by enhanced MitoSox fluorescence. Additionally, the mitochondrial membrane potential and mitochondrial ROS had a significant negative correlation (r = −0.92, 4T1.2-Luc and −0.95, MDA-MB-231), mitochondrial membrane potential and cell viability had a significant positive correlation (r = 0.99, 4T1.2-Luc and 0.81, MDA-MB-231), and mitochondrial ROS and cell viability had a significant negative correlation (r = −0.98, 4T1.2-Luc and −0.88, MDA-MB-231) (Fig. 8C, D). These results in two separate TNBC cell lines from two different species along with our previous findings [17,18] demonstrates that 9S1R NulloPT treatment alters cellular metabolism by inducing changes in mitochondrial physiology.

## 4. Discussion

This is the first in vivo study to demonstrate the physiological impact of nullomer-based peptide drugs. 9S1R-NulloPT (9S1R peptide in trehalose) was very well tolerated in vivo when delivered intraperitoneally in normal mice. Our in vivo RNAseq results support previous work showing that 9S1R-NulloPT targets metabolic pathways, specifically the genes for mitochondrial and ribosomal functions. The effect of this drug on mitochondrial physiology and ATP production has previously been demonstrated in different types of cancer cells [17,18] as well as in several TNBC cell lines. It has been reported that 9S1R-NulloPT reduces ATP formation and mitochondrial membrane potential, and increases mitochondrial reactive oxygen species (ROS) generation. This report, provides further evidence that 9S1R-NulloPT kills TNBC cells by altering cellular metabolism via the mitochondria. A moderate to mild mitochondrial stress and fission is beneficial for TNBC cell growth and aggressiveness, but severe mitochondrial stress and increased fission leads to excessive ROS generation and death of cancer cells [59]. The drastic alteration in the mitochondrial pathways in the 9S1R-NulloPT treated tumors shows that the peptides directly or indirectly target mitochondria, which could be exploited as a potential mitochondria-targeted anti-cancer/tumor therapy. Ribosome synthesis in the nucleolus increases in cancer cells to cope with increased demand for protein synthesis. Ribosome biogenesis-targeting (CX-3543, CX-5461 [60,61] and BMH [62]) is still in its infancy, however, it is emerging as an effective cancer therapy used in multiple clinical trials [63]. Biogenesis of ribosomes make an interesting target for cancer chemotherapy for many reasons: (1) the inhibition of ribosome biogenesis induces cell cycle arrest in a p53-independent manner [63], (2) these inhibitions don’t affect the resting cells, possibly due to the long half-life of cytoplasmic ribosomes [64], and (3) it could lead to apoptosis of neoplastic cells that have a high nucleolar ribosomal biogenesis rate [65]. Ribosome synthesis is extremely complex and one of the most energetically demanding cellular activities [66]. We hypothesize that the drug 9S1R-NulloPT targets mitochondrial ATP production and ribosome biogenesis leading to a loss of metabolic activity. To ensure more efficient killing of these aggressive TNBC cells in vivo and to enable tumor size reduction, we plan to use our drug synergistically along with other anticancer drugs that act through different cytotoxic pathways [67–70].

Transcriptomic analysis revealed upregulation of: unique clusters of ECM organization genes (collagen formation and degradation genes such as Col6a3, Col5a3 Plod1, Mmp3, Loxl3 and Plec); focal adhesion genes (Itga5, Lamc1, shc1, Arghap35, and Pxn), and cytoskeletal protein binding genes (Abl1, Map1a, Map1b, Map6, Myo10 and Trak1). Among the highest upregulated genes Lhx6 was noteworthy in relevance to BC, as it is reported to suppress activation of the PI3K/Akt/mTOR signaling, inhibiting the progression of BC [71]. The most downregulated genes belonged to the Igkv family, which has been suggested as an identifying biomarker for TNBC cancers [72]; and the Fcmr gene whose knockdown leads to increased phagocytosis, enhanced antigen presentation, and heightened T cell activation. Fcmr is also a promising anti-cancer target [73].

Although treatment did not change tumor size post necropsy, the in vivo as well as ex vivo BLI of the tumors showed reduced bioluminescence in the treated group. There were multiple mice (~50%) in the treatment group that showed a reduction in bioluminescence which implies fewer cells were present in the tumor due to decreased proliferation or increased cell death and loss of metabolic activity of the tumor. This corroborates the results obtained in the pilot study. Ex vivo BLI of the tumors in both the studies showed a significant decrease in bioluminescence and thus a decrease in the metabolic activity of the tumor cells in the treated groups but not in controls. This suggests that although the treatment does not change the size of tumors drastically, it altered the metabolism of the tumor cells rendering them inactive. This makes sense in light of the tumor transcriptomics: metabolism related genes were mostly downregulated in the treated tumors, specifically the mitochondrial and ribosomal genes that are essential for energy production and metabolism. The in vivo BLI from day 22 (24 h after dose 5) clearly shows the reduced bioluminescence in treated tumors during this period. In the pilot study (using a bilateral tumor model) we imaged mice after the 4th 9S1R-NulloPT dose (day 22, Supplementary Fig 4A) and found a similar decrease in in vivo bioluminescence in the treatment groups.

Histopathological evaluation confirmed that treatment and control tumors had the same grade, stage and aggressiveness. However, it was interesting to find that there was a significant change in the tumor immune-microenvironment of the treated tumors. Immune cell infiltration increased significantly in the 9S1R-NulloPT treated tumors, as measured by plasma cell numbers and margin inflammation. A positive correlation of plasma cells with favorable patient outcomes has recently been reported [43]. It is also well established that the outcome of immunotherapy treatment, and thus prognosis of BC, is dictated by the tumor microenvironment. Recent reports suggest that the immune score of BC patients could be useful for treatment decisions and survival prediction [46]: activated immune cell infiltration in tumors correlates with better prognosis [44]. Increased levels of tumor infiltrating lymphocytes (TILs) have been associated with disease-free status and overall survival rates in TNBC patients with and without any treatment. The presence of TILs in the breast tumor microenvironment can also predict responses to neoadjuvant therapy and adjuvant chemotherapy treatments, and high numbers of TILs correlate with increased pathological complete responses in TNBC [45]. We also found that the 9S1R-NulloPT treatment resulted in less externally visible necrosis as compared to untreated tumors or trehalose treated groups (data not shown). There was no conspicuous difference between the PBS control and trehalose groups (Supplementary Fig 4 A–G), suggesting that the immunological changes in the tumor microenvironment (TME) are a specific contribution of the nullomer peptide.

The results of this study should be seen in the light of the fact that the peptides were not stabilized and have a half-life in serum of about 9 min. Small peptides such as 9S1R tend to have shorter half-life in circulation, but when internalized their response could be effective immediately as we have seen in MDA-MB-231 cell lines [18]. Cellular internalization studies (Supplementary Fig 1) show that the peptide could be identified from crude lysed cytosol fraction (cytosol + mitochondria fraction) in several valence forms (2 +, 3 +, 4 +) from *m/z* 380.7 ± 0.1 when incubated with 4T1.2 cells for at least two hours. Biochemical properties of 9S1R are similar to known mitochondria penetrating peptides in terms of charge, hydrophobicity, poly arginine content [74], and the effects on mitochondrial functions [17], all of which suggests a potential for mitochondrial localization. Confocal micrographs of 4T1.2-Luc and MDA-MB-231 cells treated with fluorescently labeled 9S1R NulloPT for two hours under physiological condition (Fig. 1C) shows an initial cytosolic and seemingly endosomal localization of the peptide [30,33]. However, further colocalization studies with mitochondria across different time points are warranted to gain insights to the ultimate localization of the peptide. Existing literature supports the idea that short cell penetrating peptides reach into the mitochondria from cytosol after endosomal escape as early as 6 h or even after 24 h in vitro [30,31, 33,34]. Thus, here we confirmed that the 9S1R-NulloPT are internalized within the cells and are sequestered into other forms, which are more stable than those in serum, however, the mechanism of the 9S1R NulloPT’s uptake, metabolism, and final sub-cellular localization is still under investigation.

The Nullomer peptides are readily solubilized in the sugar trehalose, which has known anti-cancer properties [23,75]. In previous work, we used the nullomer-trehalose combination with the peptoprime 9R- and its scrambled version 9S1R to screen the NCI 60 cancer panel and the TNBC model MDA-MB-231 and found them to be effective in vitro. Another peptoprime linked to 5 arginines, did not show anticancer effects, and has been used as a negative control peptoprime [17,18]. As noted above, trehalose is reported to exhibit anti-cancer properties, and in this study, we did see some effects (Supplementary Fig 2–5). Trehalose alone had similar potency in tumor size reduction as 9S1R-NulloPT groups in study-2 (Supplementary Fig 3), but a contrasting higher than control tumor size in study-1 (Supplementary Fig 2), with no significant change in post mortem tumor size. Similar to the control group, trehalose did not show any effect on the immune cell infiltration or margin inflammation on the tumors (Supplementary Fig 4).

The 4T1.2-Luc cells have a specific tendency to metastasize into lungs and bone. We found specific metastasis to the lungs, and although there was a trend of reduced bioluminescence in the treated lungs as compared to controls, it was not statistically significant. There was also no significant change in metastatic foci counts in the lungs. These metastatic findings are still preliminary, primarily because of the large variations in the control groups.

Overall, we show a moderate level of therapeutic potential of 9S1R-NulloPT, in terms of tumor growth. In both of our in vivo studies, the treatment decreased tumor volume moderately with statistical significance during the growth phase of the tumor (day 18) seen by the fourth dose, however at the end of the experiment the control and treatment group had similar tumor weights. This suggests that 9S1R-NulloPT acts during an early therapeutic window in this TNBC model.

### 4.1. Caveats in the study and future plans

1. The efficacy of the peptide in reducing tumor volume wasn’t as large as the metabolic inhibition and this could be due to (a) the short half-life of the peptide in serum, (b) the dramatic rate of proliferation of TNBC cells that may be greater than rate of clearance of the cells from the tumor, making them necrotic and inactive but still large in size. To circumvent these challenges in the future we plan to (a) enhance peptide stability by loading the drug in an LNP or extracellular vesicle carrier (b) resect the tumor after dose 5 and continue drug administration to evaluate the tumor signatures and metastasis by in vivo BLI.
2. The RNA sequencing results revealed that mitochondrial and ribosomal genes are greatly affected by 9S1R-NulloPT treatment, but this needs to be confirmed by PCR. We plan to identify the targets more directly by the subcellular localization of the 9S1R-NulloPT.
3. To improve the therapeutic effect of the drug we plan to combine it with Doxorubicin along with 9S1R-NulloPT in in vivo TNBC models.
4. Transcriptomics analysis should be repeated with mammary tissue from normal mice, those with TNBC tumors, and those with TNBC tumors treated with 9S1R-NulloPT, in at least two different stages (day 18 and day 27).
5. We did not perform any intravenous administration as we found previously that 9S1R-NulloPT has a low but significant RBC hemolytic activity of 0.5% at 10 μM [17].

## 5. Conclusion

Previously NulloPTs showed very promising in vitro results using the NCI 60 panel of cells. The current in vivo mouse study demonstrates that the drug reduces early tumor volume (up to the 5th treatment) with no significant change in terminal tumor mass. The treatment modifies the cancer cell microenvironment by rendering it metabolically inactive, as shown by the reduced tumor bioluminescence, and previous in vitro studies and by the downregulation of metabolic pathway-related genes involved with mitochondrial function, ATP production, and ribosome assembly. These results corroborate our previous findings from the NCI 60 panel, where we have shown that the 9S1R-NulloPT leads to a drastic decrease in mitochondrial membrane potential and ATP generation, and a noticeable increase in mitochondrial ROS generation.

## Supplementary Material

1

## Figures and Tables

**Table 1-:** Functional enrichment analysis from STRING interactome: List of top five **(A)** GO terms and **(B)** pathways, based on significance of enrichment or false discovery rate (FDR) shown as a measure of p-values corrected using Benjamini–Hochberg procedure for the upregulated and downregulated DEGs.

(A) Functional enrichments in network: Top 5 GO terms based on significance FDR Upregulated genes					
	GO-term	Description	Count in network	Strength	FDR
**Biological process (Gene ontology)**	GO:0009653	Anatomical structure morphogenesis	117 of 2244	0.51	3.97E-26
	GO:0032502	Developmental process	192 of 5629	0.32	3.73E-25
	GO:0048856	Anatomical structure development	184 of 5258	0.33	4.96E-25
	GO:0007275	Multicellular organism development	176 of 4921	0.34	1.35E-24
	GO:0065007	Biological regulation	275 of 10591	0.2	1.35E-24
**Molecular function (Gene ontology)**	GO:0005515	Protein binding	204 of 6764	0.27	9.56E-21
	GO:0005488	Binding	272 of 11199	0.17	8.13E-19
	GO:0008092	Cytoskeletal protein binding	57 of 947	0.57	7.5E-14
	GO:0003779	Actin binding	35 of 418	0.71	1.3E-11
	GO:0044877	Protein-containing complex binding	65 of 1411	0.45	4.98E-11
**Cellular component Gene ontology)**	GO:0030054	Cell junction	96 of 2050	0.46	5.05E-18
	GO:0110165	Cellular anatomical entity	328 of 15632	0.11	5.05E-18
	GO:0005856	Cytoskeleton	94 of 2060	0.45	3.84E-17
	GO:0005622	Intracellular	286 of 12596	0.14	1.35E-16
	GO:0015629	Actin cytoskeleton	41 of 458	0.74	2.95E-15
**Downregulated genes Biological process (Gene ontology)**	GO:0006955	Immune response	115 of 979	0.58	6.93E-29
	GO:0002376	Immune system process	156 of 1842	0.44	4.17E-26
	GO:0051707	Response to other organism	114 of 1145	0.51	2.77E-23
	GO:0044419	Interspecies interaction between organisms	118 of 1309	0.47	4.23E-21
	GO:0006412	Translation	56 of 316	0.76	1.98E-20
**Molecular function (Gene ontology)**	GO:0003735	Structural constituent of ribosome	52 of 155	1.04	9.37E-30
	GO:0005198	Structural molecule activity	63 of 483	0.63	4.58E-17
	GO:0015078	Proton transmembrane transporter activity	20 of 113	0.76	0.00000298
	GO:0046933	Proton-transporting ATP synthase activity, rotational mechanism	8 of 16	1.21	0.00019
	GO:0003785	Actin monomer binding	9 of 24	1.09	0.00019
**Cellular component (Gene ontology)**	GO:0110165	Cellular anatomical entity	607 of 15632	0.1	6.84E-28
	GO:0005737	Cytoplasm	467 of 10283	0.17	7.46E-28
	GO:0005840	Ribosome	55 of 213	0.92	1.68E-27
	GO:0044391	Ribosomal subunit	50 of 181	0.95	4.18E-26
	GO:0022626	Cytosolic ribosome	40 of 99	1.12	8.46E-26
(B) Functional enrichments in network: Top 5 pathways based on significance FDR Upregulated genes in pathway					
	GO-term	Description	Count in network	Strength	FDR
**Biological process (Gene ontology)**	mmu04510	Focal adhesion	18 of 196	0.75	0.00000403
	mmu04360	Axon guidance	16 of 176	0.75	0.000015
	mmu04512	ECM-receptor interaction	10 of 87	0.85	0.0004
	mmu04810	Regulation of actin cytoskeleton	15 of 212	0.64	0.0004
	mmu05205	Proteoglycans in cancer	13 of 199	0.6	0.0027
**Molecular function (Gene ontology)**	MMU-1474244	Extracellular matrix organization	25 of 295	0.72	0.000000133
	MMU-162582	Signal Transduction	79 of 2417	0.3	0.00000163
	MMU-422475	Axon guidance	22 of 278	0.69	0.00000202
	MMU-9006934	Signaling by Receptor Tyrosine Kinases	25 of 418	0.57	0.0000199
	MMU-194315	Signaling by Rho GTPases	22 of 381	0.55	0.00018
**Cellular component Gene ontology)**	WP488	Alpha 6 beta 4 integrin signaling pathway	12 of 66	1.05	0.000000793
	WP85	Focal adhesion	16 of 183	0.73	0.0000146
	WP2573	Primary focal segmental glomerulosclerosis (FSGS)	10 of 72	0.93	0.0000499
	WP65	Insulin signaling	14 of 158	0.74	0.0000499
	WP6	Integrin-mediated cell adhesion	11 of 99	0.83	0.0000629
**Downregulated genes in pathway**					
**Biological process (Gene ontology)**	MMU-	SRP-dependent cotranslational protein targeting to membrane	40 of 88	1.17	1.11E-26
	1799339				
	MMU-975956	Nonsense Mediated Decay (NMD) independent of the Exon Junction Complex (EJC)	39 of 90	1.15	1.08E-25
	MMU-72689	Formation of a pool of free 40 S subunits	40 of 97	1.13	1.08E-25
	MMU-156827	L13a-mediated translational silencing of Ceruloplasmin expression	41 of 107	1.1	1.08E-25
	MMU-72706	GTP hydrolysis and joining of the 60 S ribosomal subunit	41 of 108	1.09	1.08E-25
**Molecular function (Gene ontology)**	MMU-1799339	SRP-dependent cotranslational protein targeting to membrane	40 of 88	1.17	1.11E-26
	MMU-975956	Nonsense Mediated Decay (NMD) independent of the Exon Junction Complex (EJC)	39 of 90	1.15	1.08E-25
	MMU-72689	Formation of a pool of free 40 S subunits	40 of 97	1.13	1.08E-25
	MMU-156827	L13a-mediated translational silencing of Ceruloplasmin expression	41 of 107	1.1	1.08E-25
	MMU-72706	GTP hydrolysis and joining of the 60 S ribosomal subunit	41 of 108	1.09	1.08E-25
**Cellular component (Gene ontology)**	WP163	Cytoplasmic ribosomal proteins	35 of 77	1.17	5.24E-24
	WP295	Electron transport chain	36 of 97	1.08	1.34E-22
	WP1248	Oxidative phosphorylation	26 of 58	1.16	6.23E-18
	WP1253	Type II interferon signaling (IFNG)	10 of 32	1.01	0.0000161
	WP2271	Macrophage markers	5 of 10	1.21	0.0022

## Data Availability

Data will be made available on request.

## List of abbreviations:

9S1R NulloPT: 9S1R nullomer peptide in trehalose
aa: amino acid
BC: Breast cancer
BLI: Bioluminescence imaging
DEGs: Differentially expressed genes
ECM: Extracellular matrix
EIC: Extracted ion chromatogram
ERɑ: Estrogen receptor alpha
ETC: Electron transport chain
FDR: False discovery rate
GO: Gene ontology
H&E: Hematoxylin and Eosin
HER: Human epidermal growth factor receptor
IP: Intraperitoneal
IVIS: In vivo imaging system
MS: Mass Spectrometry
mTNBC: metastatic TNBC
MTP: Mitochondria penetrating peptide
MTT: 3-(4,5-dimethylthiazol-2-yl)–2,5-diphenyltetrazolium bromide
NulloP: Nullomer Peptide
NulloPT: Nullomer peptide in trehalose
Padj: P adjusted values
PD 1: Programmed cell death protein-1
PDL1: Programmed cell death Ligand-1
PR: Progesterone receptors
ROS: Reactive Oxygen Species
SEER: Surveillance, Epidemiology, and End Results
TCA: Tri Carboxylic Acid Cycle
TIL: Tumor infiltrating lymphocyte
TME: Tumor microenvironment
TMRM: Tetramethylrhodamine, Methyl Ester, Perchlorate
TNBC: Triple Negative Breast Cancee

## References

[1] Siegel RL, Miller KD, Fuchs HE, Jemal A, Cancer Statistics, 2021, CA Cancer J. Clin 71 (1) (2021) 7–33. 33433946 10.3322/caac.21654

[2] American Cancer Society. Cancer Facts & Figures, Atlanta: American Cancer, Society 2023 (2023) 11. 10.6004/jadpro.2020.11.2.1 PMC7848816 33532112

[3] Global Cancer Observatory International Agency for Research on Cancer 2021 [cited 2021 September 22]. Available from: http://gco.iarc.fr/.).

[4] Kadamkulam Syriac A, Nandu NS, Leone JP, Central nervous system metastases from triple-negative breast cancer: current treatments and future prospective, Breast Cancer 14 (2022) 1–13. 35046721 10.2147/BCTT.S274514 PMC8760391

[5] American Cancer Society, Breast Cancer. Facts & Figures 2017–2019, American Cancer Society, Atlanta, 2017.

[6] Toulouie S, Johanning G, Shi Y, Chimeric antigen receptor T-cell immunotherapy in breast cancer: development and challenges, J. Cancer 12 (4) (2021) 1212–1219. 33442419 10.7150/jca.54095 PMC7797648

[7] FDA approves pembrolizumab for high-risk early-stage triple-negative breast cancer 2021 [cited 2021 September 20]. Available from: https://www.fda.gov/drugs/resources-information-approved-drugs/fda-approves-pembrolizumab-high-risk-early-stage-triple-negative-breast-cancer.

[8] Carey LA, Loirat D, Punie K, Bardia A, Dieras V, Dalenc F, Diamond JR, Fontaine C, Wang G, Rugo HS, , Sacituzumab govitecan as second-line treatment for metastatic triple-negative breast cancer-phase 3 ASCENT study subanalysis, NPJ Breast Cancer 8 (1) (2022), 72. 35680967 10.1038/s41523-022-00439-5 PMC9184615

[9] FDA Approves New Therapy for Triple Negative Breast Cancer That Has Spread, Not Responded to Other Treatments. 2020.

[10] Mittendorf EA Hunt KK Breast Cancer Immunotherapy: Is It Ready for Prime Time? In 2015 2015.

[11] Mehrotra N, Kharbanda S, Singh H, Peptide-based combination nanoformulations for cancer therapy, Nanomedicine 15 (22) (2020) 2201–2217. 32914691 10.2217/nnm-2020-0220

[12] Xie M, Liu D, Yang Y, Anti-cancer peptides: classification, mechanism of action, reconstruction and modification, Open Biol. 10 (7) (2020), 200004. 32692959 10.1098/rsob.200004 PMC7574553

[13] Karami Fath M, Babakhaniyan K, Zokaei M, Yaghoubian A, Akbari S, Khorsandi M, Soofi A, Nabi-Afjadi M, Zalpoor H, Jalalifar F, , Anti-cancer peptide-based therapeutic strategies in solid tumors, Cell Mol. Biol. Lett 27 (1) (2022), 33. 35397496 10.1186/s11658-022-00332-w PMC8994312

[14] Hampikian G, Andersen T, Absent sequences: nullomers and primes, Pac. Symp. Biocomput (2007) 355–366. 17990505 10.1142/9789812772435_0034

[15] Georgakopoulos-Soares I, Yizhar-Barnea O, Mouratidis I, Hemberg M, Ahituv N, Absent from DNA and protein: genomic characterization of nullomers and nullpeptides across functional categories and evolution, Genome Biol. 22 (1) (2021), 245. 34433494 10.1186/s13059-021-02459-z PMC8386077

[16] Mouratidis I, Chan CSY, Chantzi N, Tsiatsianis GC, Hemberg M, Ahituv N, Georgakopoulos-Soares I, Quasi-prime peptides: identification of the shortest peptide sequences unique to a species, NAR Genom. Bioinform 5 (2) (2023), lqad039. 37101657 10.1093/nargab/lqad039 PMC10124967

[17] Alileche A, Goswami J, Bourland W, Davis M, Hampikian G, Nullomer derived anticancer peptides (NulloPs): differential lethal effects on normal and cancer cells in vitro, Peptides 38 (2) (2012) 302–311. 23000474 10.1016/j.peptides.2012.09.015

[18] Alileche A, Hampikian G, The effect of Nullomer-derived peptides 9R, 9S1R and 124R on the NCI-60 panel and normal cell lines, BMC Cancer 17 (1) (2017), 533. 28793867 10.1186/s12885-017-3514-z PMC5551024

[19] Goswami J, Davis MC, Andersen T, Alileche A, Hampikian G, Safeguarding forensic DNA reference samples with nullomer barcodes, J. Forensic Leg. Med 20 (5) (2013) 513–519. 23756524 10.1016/j.jflm.2013.02.003

[20] Bumbat M, Wang M, Liang W, Ye P, Sun W, Liu B, Effects of Me(2)SO and Trehalose on the Cell Viability, Proliferation, and Bcl-2 Family Gene (BCL-2, BAX, and BAD) Expression in Cryopreserved Human Breast Cancer Cells, Biopreserv Biobank 18 (1) (2020) 33–40. 31800305 10.1089/bio.2019.0082

[21] Hirano R, Kagamiya T, Matsumoto Y, Furuta T, Sakurai M, Molecular mechanism underlying the selective attack of trehalose lipids on cancer cells as revealed by coarse-grained molecular dynamics simulations, Biochem. Biophys. Rep 25 (2021), 100913. 33521337 10.1016/j.bbrep.2021.100913 PMC7820381

[22] Jiang YL, Li SX, Liu YJ, Ge LP, Han XZ, Liu ZP, Synthesis and evaluation of trehalose-based compounds as novel inhibitors of cancer cell migration and invasion, Chem. Biol. Drug Des 86 (5) (2015) 1017–1029. 25855371 10.1111/cbdd.12569

[23] Devi NSNC, Sahu A, Alugoju S, P: Molecular mechanisms of action of Trehalose in cancer: a comprehensive review, Life Sci. 269 (2021), 118968. 33417959 10.1016/j.lfs.2020.118968

[24] Nikolova B, Antov G, Semkova S, Tsoneva I, Christova N, Nacheva L, Kardaleva P, Angelova S, Stoineva I, Ivanova J, , Bacterial Natural Disaccharide (Trehalose Tetraester): molecular modeling and in vitro study of anticancer activity on breast cancer cells, Polymers 12 (2) (2020). 10.3390/polym12020499 PMC7077702 32102469

[25] Bolin C, Sutherland C, Tawara K, Moselhy J, Jorcyk CL, Novel mouse mammary cell lines for in vivo bioluminescence imaging (BLI) of bone metastasis, Biol. Proced. Online 14 (1) (2012), 6. 22510147 10.1186/1480-9222-14-6 PMC3473320

[26] Dutta D, Ali N, Banerjee E, Singh R, Naskar A, Paidi RK, Mohanakumar KP, Low levels of prohibitin in substantia nigra makes dopaminergic neurons vulnerable in Parkinson’s Disease, Mol. Neurobiol 55 (1) (2018) 804–821. 28062948 10.1007/s12035-016-0328-y

[27] Farhadian M, Rafat SA, Panahi B, Ebrahimie E, Transcriptome signature of two lactation stages in Ghezel sheep identifies using RNA-Sequencing, Anim. Biotechnol 33 (2) (2022) 223–233. 32633600 10.1080/10495398.2020.1784185

[28] Appukuttan TA, Ali N, Varghese M, Singh A, Tripathy D, Padmakumar M, Gangopadhyay PK, Mohanakumar KP, Parkinson’s disease cybrids, differentiated or undifferentiated, maintain morphological and biochemical phenotypes different from those of control cybrids, J. Neurosci. Res 91 (7) (2013) 963–970. 23653325 10.1002/jnr.23241

[29] Ali N, Sane MS, Tang H, Compher J, McLaughlin Q, Jones CD, Maffi SK, 6-hydroxydopamine affects multiple pathways to induce cytotoxicity in differentiated LUHMES dopaminergic neurons, Neurochem. Int 170 (2023), 105608. 37678429 10.1016/j.neuint.2023.105608

[30] Oh D, Nasrolahi Shirazi A, Northup K, Sullivan B, Tiwari RK, Bisoffi M, Parang K, Enhanced cellular uptake of short polyarginine peptides through fatty acylation and cyclization, Mol. Pharm 11 (8) (2014) 2845–2854. 24978295 10.1021/mp500203e PMC4144761

[31] Uusna J, Langel K, Langel U, Toxicity, immunogenicity, uptake, and kinetics methods for CPPs, Methods Mol. Biol 1324 (2015) 133–148. 26202267 10.1007/978-1-4939-2806-4_9

[32] Ohgita T, Takechi-Haraya Y, Okada K, Matsui S, Takeuchi M, Saito C, Nishitsuji K, Uchimura K, Kawano R, Hasegawa K, , Enhancement of direct membrane penetration of arginine-rich peptides by polyproline II helix structure, Biochim Biophys. Acta Biomembr 1862 (10) (2020), 183403. 32585206 10.1016/j.bbamem.2020.183403

[33] Wallbrecher R, Ackels T, Olea RA, Klein MJ, Caillon L, Schiller J, Bovee-Geurts PH, van Kuppevelt TH, Ulrich AS, Spehr M, , Membrane permeation of arginine-rich cell-penetrating peptides independent of transmembrane potential as a function of lipid composition and membrane fluidity, J. Control Release 256 (2017) 68–78. 28411183 10.1016/j.jconrel.2017.04.013

[34] Allolio C, Magarkar A, Jurkiewicz P, Baxova K, Javanainen M, Mason PE, Sachl R, Cebecauer M, Hof M, Horinek D, , Arginine-rich cell-penetrating peptides induce membrane multilamellarity and subsequently enter via formation of a fusion pore, Proc. Natl. Acad. Sci 115 (47) (2018) 11923–11928. 30397112 10.1073/pnas.1811520115 PMC6255155

[35] Chamberlain GR, Tulumello DV, Kelley SO, Targeted delivery of doxorubicin to mitochondria, ACS Chem. Biol 8 (7) (2013) 1389–1395. 23590228 10.1021/cb400095v

[36] Jean SR, Ahmed M, Lei EK, Wisnovsky SP, Kelley SO, Peptide-mediated delivery of chemical probes and therapeutics to mitochondria, Acc. Chem. Res 49 (9) (2016) 1893–1902. 27529125 10.1021/acs.accounts.6b00277

[37] Jean SR, Pereira MP, Kelley SO, Structural modifications of mitochondria-targeted chlorambucil alter cell death mechanism but preserve MDR evasion, Mol. Pharm 11 (8) (2014) 2675–2682. 24922525 10.1021/mp500104j

[38] Yousif LF, Stewart KM, Horton KL, Kelley SO, Mitochondria-penetrating peptides: sequence effects and model cargo transport, Chembiochem 10 (12) (2009) 2081–2088. 19670199 10.1002/cbic.200900017

[39] Yousif LF, Stewart KM, Kelley SO, Targeting mitochondria with organelle-specific compounds: strategies and applications, Chembiochem 10 (12) (2009) 1939–1950. 19637148 10.1002/cbic.200900185

[40] Enot DP, Vacchelli E, Jacquelot N, Zitvogel L, Kroemer G, TumGrowth: an open-access web tool for the statistical analysis of tumor growth curves, Oncoimmunology 7 (9) (2018), e1462431. 30228932 10.1080/2162402X.2018.1462431 PMC6140814

[41] Ji X, Adams ST Jr, Miller SC, Bioluminescence imaging in mice with synthetic luciferin analogues, Methods Enzym. 640 (2020) 165–183. 10.1016/bs.mie.2020.04.033 PMC8345814 32560797

[42] Ghasemi M, Turnbull T, Sebastian S, Kempson I, The MTT Assay: utility, limitations, pitfalls, and interpretation in bulk and single-cell analysis, Int J. Mol. Sci 22 (23) (2021). 10.3390/ijms222312827 PMC8657538 34884632

[43] Sakaguchi A, Horimoto Y, Onagi H, Ikarashi D, Nakayama T, Nakatsura T, Shimizu H, Kojima K, Yao T, Matsumoto T, , Plasma cell infiltration and treatment effect in breast cancer patients treated with neoadjuvant chemotherapy, Breast Cancer Res. 23 (1) (2021), 99. 34715905 10.1186/s13058-021-01477-w PMC8555250

[44] Lv Y, Lv D, Lv X, Xing P, Zhang J, Zhang Y, Immune cell infiltration-based characterization of triple-negative breast cancer predicts prognosis and chemotherapy response markers, Front. Genet 12 (2021), 616469. 33815462 10.3389/fgene.2021.616469 PMC8017297

[45] Garcia-Teijido P, Cabal ML, Fernandez IP, Perez YF, Tumor-infiltrating lymphocytes in triple negative breast cancer: the future of immune targeting, Clin. Med. Insights Oncol 10 (Suppl 1) (2016) 31–39. 27081325 10.4137/CMO.S34540 PMC4822722

[46] Wang S, Zhang Q, Yu C, Cao Y, Zuo Y, Yang L, Immune cell infiltration-based signature for prognosis and immunogenomic analysis in breast cancer, Brief. Bioinform 22 (2) (2021) 2020–2031. 32141494 10.1093/bib/bbaa026

[47] Zhang L, Zhang W, Chen J, Regulatory mechanism of immune-related genes in patients with hypertension, Medicine 102 (9) (2023), e32627. 36862882 10.1097/MD.0000000000032627 PMC9981365

[48] Zhai Y, Liu X, Huang Z, Zhang J, Stalin A, Tan Y, Zhang F, Chen M, Shi R, Huang J, , Data mining combines bioinformatics discover immunoinfiltration-related gene SERPINE1 as a biomarker for diagnosis and prognosis of stomach adenocarcinoma, Sci. Rep 13 (1) (2023), 1373. 36697459 10.1038/s41598-023-28234-7 PMC9876925

[49] Schrors B, Boegel S, Albrecht C, Bukur T, Bukur V, Holtstrater C, Ritzel C, Manninen K, Tadmor AD, Vormehr M, , Multi-Omics Characterization of the 4T1 Murine Mammary Gland Tumor Model, Front. Oncol 10 (2020), 1195. 32793490 10.3389/fonc.2020.01195 PMC7390911

[50] Eckhardt BL, Parker BS, van Laar RK, Restall CM, Natoli AL, Tavaria MD, Stanley KL, Sloan EK, Moseley JM, Anderson RL, Genomic analysis of a spontaneous model of breast cancer metastasis to bone reveals a role for the extracellular matrix, Mol. Cancer Res 3 (1) (2005) 1–13. 15671244

[51] Kim G-E, Kim NI, Lee JS, Park MH, Kang K, Differentially expressed genes in matched normal, Cancer, and Lymph Node Metastases Predict Clinical Outcomes in Patients With Breast Cancer, Appl. Immunohistochem. Mol. Morphol 28 (2) (2020) 111–122. 32044879 10.1097/PAI.0000000000000717 PMC7028469

[52] Kaur J, Chandrashekar DS, Varga Z, Sobottka B, Janssen E, Kowalski J, Kiraz U, Varambally S, Aneja R, Distinct Gene Expression Profiles of Matched Primary and Metastatic Triple-Negative Breast Cancers, Cancers 14 (10) (2022). 10.3390/cancers14102447 PMC9139196 35626050

[53] Chen J, Gao G, Li L, Ding J, Chen X, Lei J, Long H, Wu L, Long X, He L, , Pan-Cancer Study of SHC-Adaptor Protein 1 (SHC1) as a Diagnostic, Prognostic and Immunological Biomarker in Human Cancer, Front. Genet 13 (2022), 817118. 35601500 10.3389/fgene.2022.817118 PMC9115805

[54] Zhang Y, Kwok-Shing Ng P, Kucherlapati M, Chen F, Liu Y, Tsang YH, de Velasco G, Jeong KJ, Akbani R, Hadjipanayis A, , A Pan-Cancer Proteogenomic Atlas of PI3K/AKT/mTOR Pathway Alterations, Cancer Cell 31 (6) (2017) 820–832, e823. 28528867 10.1016/j.ccell.2017.04.013 PMC5502825

[55] Raisch J, Cote-Biron A, Rivard N, A Role for the WNT Co-Receptor LRP6 in Pathogenesis and Therapy of Epithelial Cancers, Cancers 11 (8) (2019). 10.3390/cancers11081162 PMC6721565 31412666

[56] Jiang W, Crossman DK, Mitchell EH, Sohn P, Crowley MR, Serra R, WNT5A inhibits metastasis and alters splicing of Cd44 in breast cancer cells, PLoS One 8 (3) (2013), e58329. 23484019 10.1371/journal.pone.0058329 PMC3590134

[57] Morrison CD, Chang JC, Keri RA, Schiemann WP, Mutant p53 dictates the oncogenic activity of c-Abl in triple-negative breast cancers, Cell Death Dis. 8 (6) (2017), e2899. 28661474 10.1038/cddis.2017.294 PMC5520943

[58] Chen F, Han B, Meng Y, Han Y, Liu B, Zhang B, Chang Y, Cao P, Fan Y, Tan K, Ceruloplasmin correlates with immune infiltration and serves as a prognostic biomarker in breast cancer, Aging 13 (16) (2021) 20438–20467. 34413268 10.18632/aging.203427 PMC8436892

[59] Weiner-Gorzel K, Murphy M, Mitochondrial dynamics, a new therapeutic target for Triple Negative Breast Cancer, Biochim Biophys. Acta Rev. Cancer 1875 (2) (2021), 188518. 33545296 10.1016/j.bbcan.2021.188518

[60] Bywater MJ, Poortinga G, Sanij E, Hein N, Peck A, Cullinane C, Wall M, Cluse L, Drygin D, Anderes K, , Inhibition of RNA polymerase I as a therapeutic strategy to promote cancer-specific activation of p53, Cancer Cell 22 (1) (2012) 51–65. 22789538 10.1016/j.ccr.2012.05.019 PMC3749732

[61] Drygin D, Lin A, Bliesath J, Ho CB, O’Brien SE, Proffitt C, Omori M, Haddach M, Schwaebe MK, Siddiqui-Jain A, , Targeting RNA polymerase I with an oral small molecule CX-5461 inhibits ribosomal RNA synthesis and solid tumor growth, Cancer Res. 71 (4) (2011) 1418–1430. 21159662 10.1158/0008-5472.CAN-10-1728

[62] Peltonen K, Colis L, Liu H, Trivedi R, Moubarek MS, Moore HM, Bai B, Rudek MA, Bieberich CJ, Laiho M, A targeting modality for destruction of RNA polymerase I that possesses anticancer activity, Cancer Cell 25 (1) (2014) 77–90. 24434211 10.1016/j.ccr.2013.12.009 PMC3930145

[63] Penzo M, Montanaro L, Trere D, Derenzini M, The Ribosome Biogenesis-Cancer Connection, Cells 8 (1) (2019). 10.3390/cells8010055 PMC6356843 30650663

[64] Nikolov EN, Dabeva MD, Nikolov TK, Turnover of ribosomes in regenerating rat liver, Int. J. Biochem 15 (10) (1983) 1255–1260. 6628827 10.1016/0020-711x(83)90215-x

[65] Scala F, Brighenti E, Govoni M, Imbrogno E, Fornari F, Trere D, Montanaro L, Derenzini M, Direct relationship between the level of p53 stabilization induced by rRNA synthesis-inhibiting drugs and the cell ribosome biogenesis rate, Oncogene 35 (8) (2016) 977–989. 25961931 10.1038/onc.2015.147

[66] Thomson E, Ferreira-Cerca S, Hurt E, Eukaryotic ribosome biogenesis at a glance, J. Cell Sci 126 (Pt 21) (2013) 4815–4821. 24172536 10.1242/jcs.111948

[67] Pelletier J, Thomas G, Volarevic S, Ribosome biogenesis in cancer: new players and therapeutic avenues, Nat. Rev. Cancer 18 (1) (2018) 51–63. 29192214 10.1038/nrc.2017.104

[68] Lin Z, Peng R, Sun Y, Zhang L, Zhang Z, Identification of ribosomal protein family in triple-negative breast cancer by bioinformatics analysis, Biosci. Rep 41 (1) (2021). 10.1042/BSR20200869 PMC7789804 33305312

[69] Catez F, Dalla Venezia N, Marcel V, Zorbas C, Lafontaine DLJ, Diaz JJ, Ribosome biogenesis: an emerging druggable pathway for cancer therapeutics, Biochem Pharmacol. 159 (2019) 74–81. 30468711 10.1016/j.bcp.2018.11.014

[70] Yu J, Wang L, Ling Y, Xiao X, Gong J, Jin H, Xu J, Chen P, Xie X, Zhang L, Peptide-modified bioresponsive chondroitin sulfate micelles for targeted doxorubicin delivery in triple-negative breast cancer, Colloids Surf. B Biointerfaces 227 (2023), 113381. 37257299 10.1016/j.colsurfb.2023.113381

[71] Bi QJ, Men XJ, Han R, Li GL, LHX6 inhibits the proliferation, invasion and migration of breast cancer cells by modulating the PI3K/Akt/mTOR signaling pathway, Eur. Rev. Med. Pharm. Sci 22 (10) (2018) 3067–3073. 10.26355/eurrev_201805_15066 29863252

[72] Huang QY, Liu GF, Qian XL, Tang LB, Huang QY, Xiong LX, Long Non-Coding RNA: dual effects on breast cancer metastasis and clinical applications, Cancers 11 (11) (2019). 10.3390/cancers11111802 PMC6896003 31744046

[73] Kubli SP, Vornholz L, Duncan G, Zhou W, Ramachandran P, Fortin J, Cox M, Han S, Nechanitzky R, Nechanitzky D, , Fcmr regulates mononuclear phagocyte control of anti-tumor immunity, Nat. Commun 10 (1) (2019) 2678. 31213601 10.1038/s41467-019-10619-w PMC6581943

[74] Horton KL, Stewart KM, Fonseca SB, Guo Q, Kelley SO, Mitochondria-penetrating peptides, Chem. Biol 15 (4) (2008) 375–382. 18420144 10.1016/j.chembiol.2008.03.015

[75] Ichihara H, Kuwabara K, Matsumoto Y, Trehalose liposomes suppress the growth of tumors on human lung carcinoma-bearing mice by induction of apoptosis in vivo, Anticancer Res 37 (11) (2017) 6133–6139. 29061794 10.21873/anticanres.12062

