# Supplementary material for: 9S1R nullomer peptide induces mitochondrial pathology, metabolic suppression, and enhanced immune cell infiltration, in triple-negative breast cancer mouse model
Source: Biomed Pharmacother. Author manuscript; Available in PMC 2024 Feb 16. (PMC10872342; doi:10.1016/j.biopha.2023.115997)
Supplement: 1 [file NIHMS1957137-supplement-1.docx]

**Supplementary information:**

**Supplementary Fig 1:** Detection of peptide internalization and stability within cells. (A) LC-MS Graph showing peak of peptide only spike at 1mg/ml final concentration (positive control) (B) Sample-1: 2 × 10^5^ 4T1.2 cells lysate was filtered through a 3KDa cut-off Amicon protein filters (Millipore) to get rid of proteins larger than 3KDa, followed by LC-MS detection (negative control). (C) Sample-2: 2 × 10^5^ 4T1.2 cells lysate (50ul) was mixed with the peptide at 1mg/ml final concentration and incubated for 30 mins followed by filtering through 3 KDa cut-off filter and LC-MS detection. (D) Sample-3: 50 ul of filtered cell lysate or Sample-1 was mixed with the peptide at 1mg/ml final concentration and incubated for 30 mins followed by LC-MS detection. (E) Sample-4: Cells were treated with 50uM of peptide and incubated for 2h followed by washing in PBS, lysis, passing through 3KDa protein filter, and detection by LC-MS. (F) The extracted ion chromatogram (EIC) peaks of the peptide only positive control from - A with retention time (X-axis) vs peak intensity (Y-axis) showing the peptide (molecular weight 1518.80 Da) in 2+ (760.39 Da), 3+ (507.2637 Da) and 4+ (380.7002 Da) valance forms (red circles). (G) Enlarged peaks from- C, Sample-2 with (H) extracted ion chromatogram (EIC) peaks of the detected peptide at the exact 3+ and 4+ valance forms (red circles). (I) Enlarged peaks from- E, Sample-4 with (J) extracted ion chromatogram (EIC) peaks of the detected peptide at the exact 3+ and 4+ valance forms (red circles). Sample -1(B) did not show similar peaks, which matches with the peptide’s valance forms or m/z values and Sample-3 (D) showed the peptide peak only. Thus, the peptide was detected inside cells in two forms.


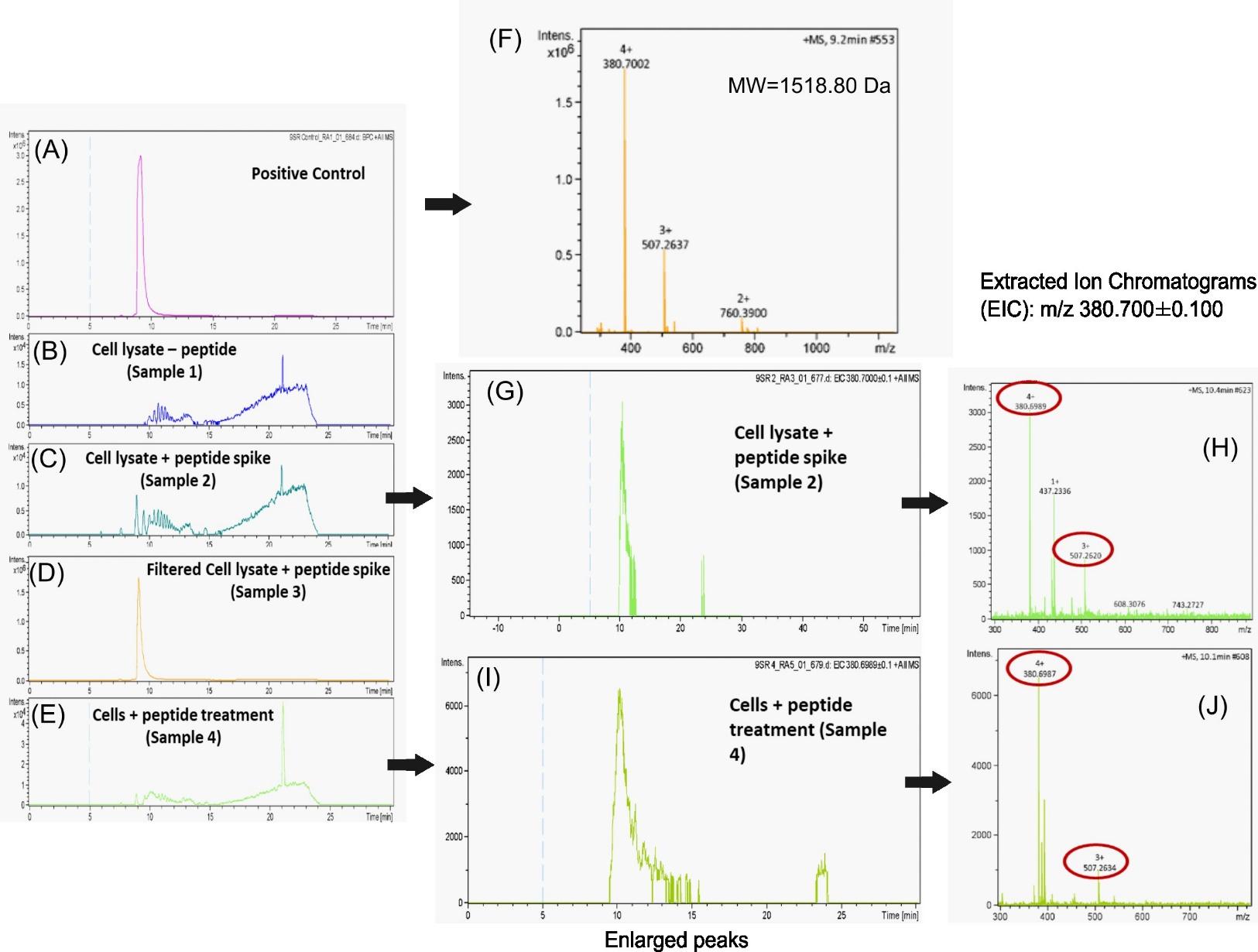


**Supplementary Fig 2:** Detailed results from pilot first study with six IP administration of PBS (control), Trehalose, 50mg/kg NulloPT and 100 mg/kg NulloPT in TNBC mice. (A) Timeline for the first in vivo pilot study. TNBC model was created by orthotopically injecting 4T1.2-Luc cells bilaterally in the 4th mammary fat pads of Balb/c mice, with 1x10^5^ cells per side. The 4T1.2-Luc cell line was developed by Dr. Cheryl Jorcyk and they express luciferase gene which serves as a luminescent indicator of gene expression or tumorigenesis, making the tumor cell traceable in vivo. Treatment started after the formation of palpable tumors and was detected by an in vivo BLI system (IVIS, Perkin Elmer) followed by randomization of the animals per group. The treatment group received IP injections of NulloPT (50 mg/kg, or 100 mg/kg), control group received PBS and Trehalose (2.5M/kg). A total of six doses per mouse was given over a period of two weeks in four mice per group. The animals were euthanized after 29 days post-tumor cell transplantation (B) The tumor volumes from both right and left tumors shows a trend of reduction in the NulloPT treated tumors when compared to PBS or Trehalose, though not statistically significant. The higher dose NulloPT group (100mg/kg) showed the best reduction in tumor size in comparison to the low dose group (50mg/kg). (C) There was a drop in the body weight following the initial treatments in the first week, which later stabilized returning to near-control weight, the loss in weight however, was not greater than 15% of the initial body weights, ruling out any adverse effect. (D) In vivo BLI at Day 15, 22 and 29 at 10s exposure. NulloPT treated mice showed a lack of signal from the tumors from day 22 onwards. (E) Images of the post mortem excised tumor and ex-vivo BLI signals from Right (R) and Left (L) tumors. Total emission measured as Photons/sec from the ex vivo BLI of (F) right tumors and (G) left tumors show a significant reduction in bioluminescence at both the treated tumors when compared to PBS (*, p<0.05) or Trehalose (@, p<0.05). (H) Ex vivo BLI showing signal from secondary lung metastasis. (I) Total metastasis counts from ex vivo BLI and necropsy show a reduction in metastasis at p<0.06 in the highest dose NulloPT (100 mg/kg) group.


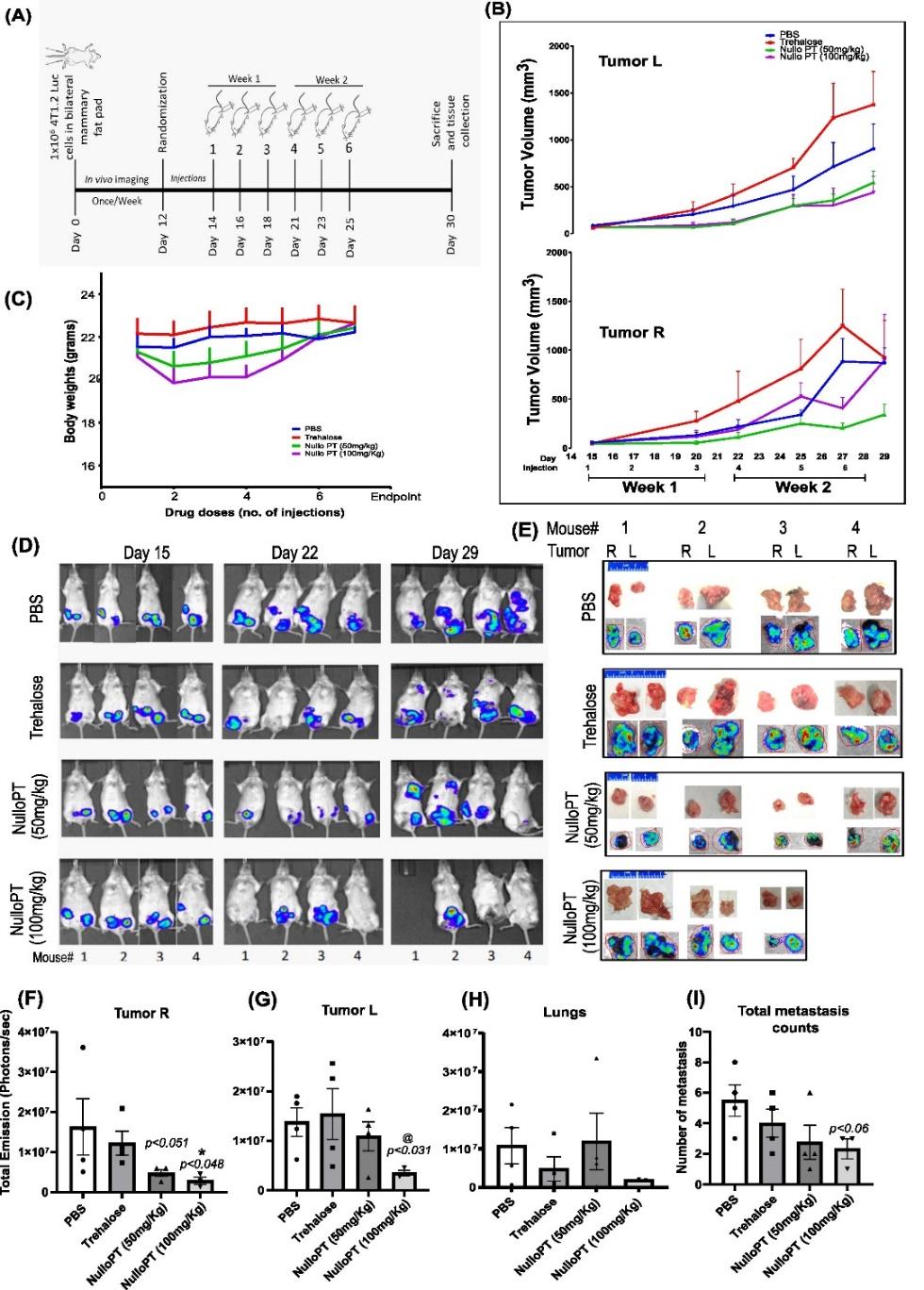


**Supplementary Fig 3:** Detailed results from second study with eight IP administration of PBS (control), Trehalose, 50mg/kg NulloPT and 100 mg/kg NulloPT in TNBC mice. (A) Images from excised tumors at endpoint from all the groups; blue scale in inches. (B) Tumor volume by caliper measurement plotted against days post tumor cell injection and injection timeline of 8 doses. (C) Change in excised tumor weight post mortem, revealed no significant difference among group (D) Body weight with time in all the groups. Data presented as Mean+ SEM followed by two-tailed unpaired Student’s t-test, *p<0.05 considered statistically significant.


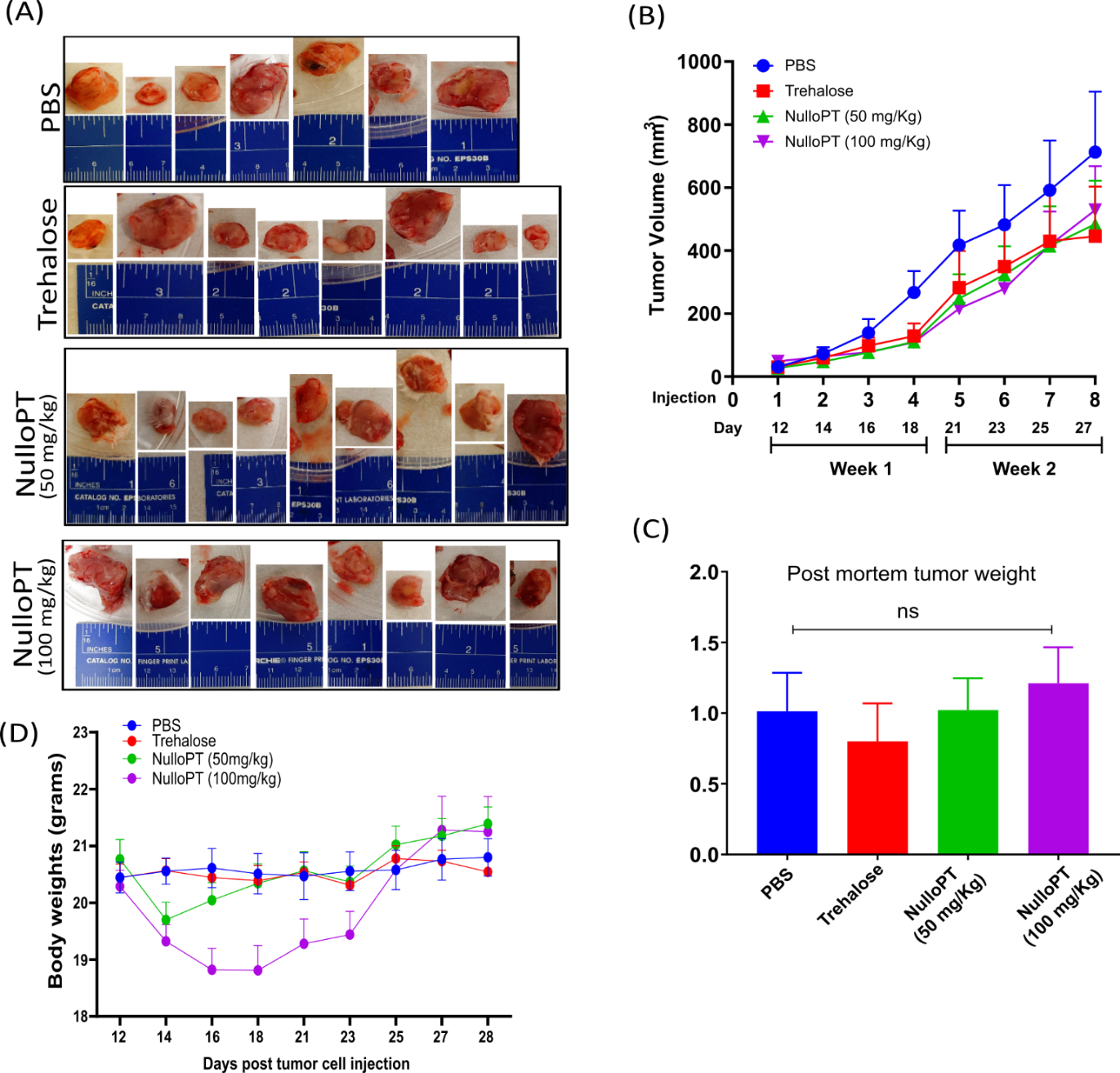


**Supplementary Fig 4:** Tumor histopathology and changes in tumor immune-microenvironment (detailed results from second study): Pathological scoring from the Hematoxylin and Eosin stained sections of the mammary tumor from PBS (control), Trehalose, 50mg/kg NulloPT and 100 mg/kg NulloPT (treated) mice shows (A) aggressiveness of the tumor, (B) stage of tumor, (C) tumor grade, (D) score of observed necrosis, (E) infiltration of immune cells inside the tumor and (F) marginal inflammation of tumor. (G) Representative image from the sections, showing increased immune cell infiltration in the NulloPT treated tumor (yellow arrows). Data represented as Mean ± SEM, Two-tailed student’s t test with * P ≤ 0.05, N=5-6. One-way Anova followed by multiple comparison post hoc analysis. Data represented as Mean ± SEM, * P ≤ 0.05, ** P ≤ 0.01, *** P ≤ 0.001, **** P ≤ 0.0001, N=5-6.


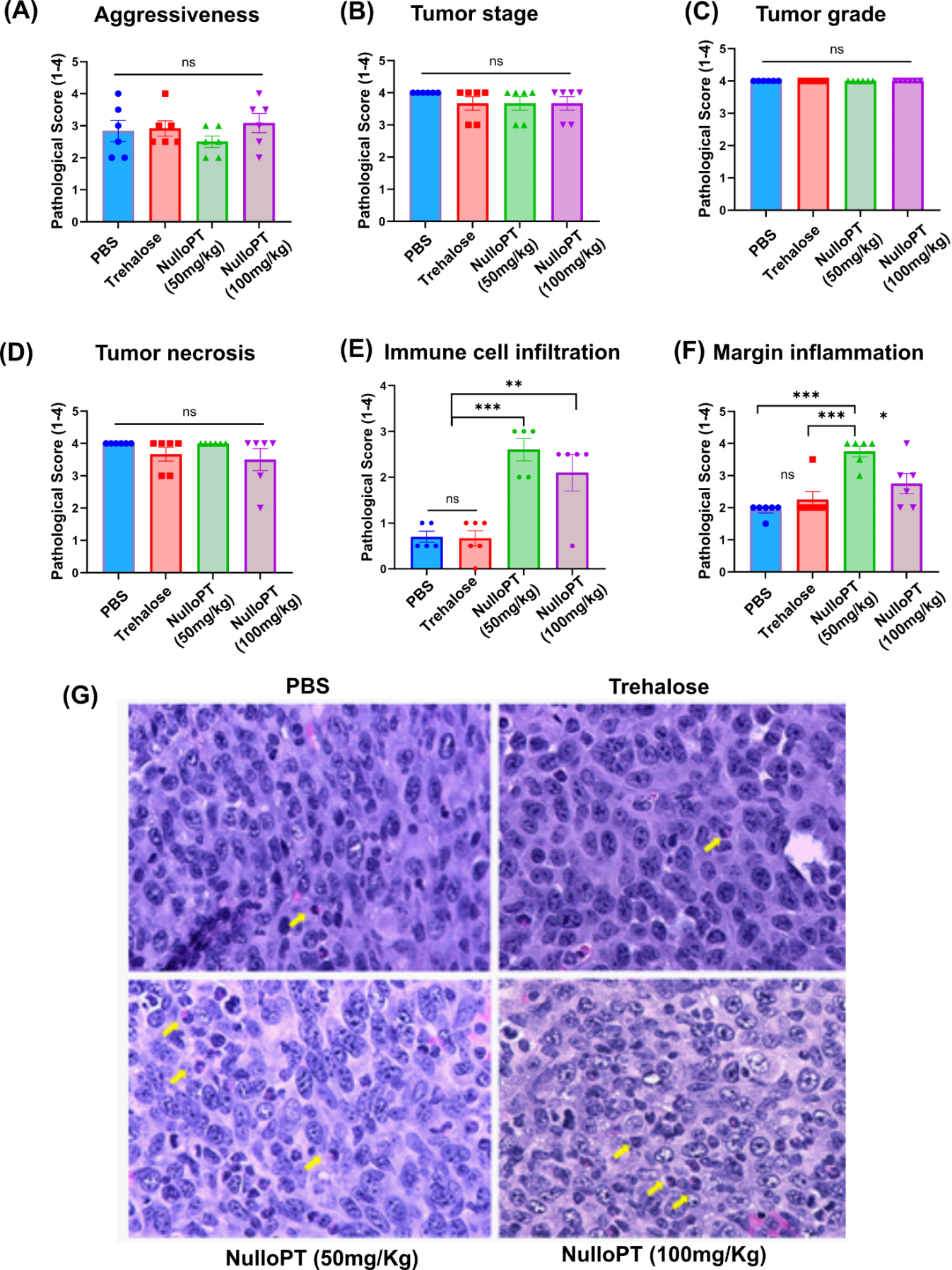


**Supplementary Fig 5:**  Comparative analysis of cancer pathways in NulloPT-treated and untreated TNBC. Comparison of upregulated DEGs obtained from NulloPT treated TNBC tumors vs untreated TNBC tumors (Fig. 7, group-a) vs, published DEGs comparing mouse 4T1 TNBC cells vs mouse mammary tissue (Fig. 7, group-b) revealed (A) The 20 upregulated cancer related DEGs indicating the effects of NulloPT, with corresponding log2 fold change and Padj values, (B) interactome of the NulloPT-treatment upregulated DEGs and (C) Upregulated NulloPT-treatment cancer pathways from Kegg, Reactome and Wikipathways with the number of genes involved within parenthesis and color code relating to the interactome of genes depicted in B. (D) The 44 common upregulated cancer-related DEGs in untreated TNBC, with corresponding log2 fold change and Padj values, (E) Untreated TNBC PPI interactome with nodes depicting the upregulated DEGs and (F) Untreated TNBC cancer pathways from Kegg, Reactome and Wikipathways with the number of genes involved within parenthesis and color code relating to the interactome of genes at-E.


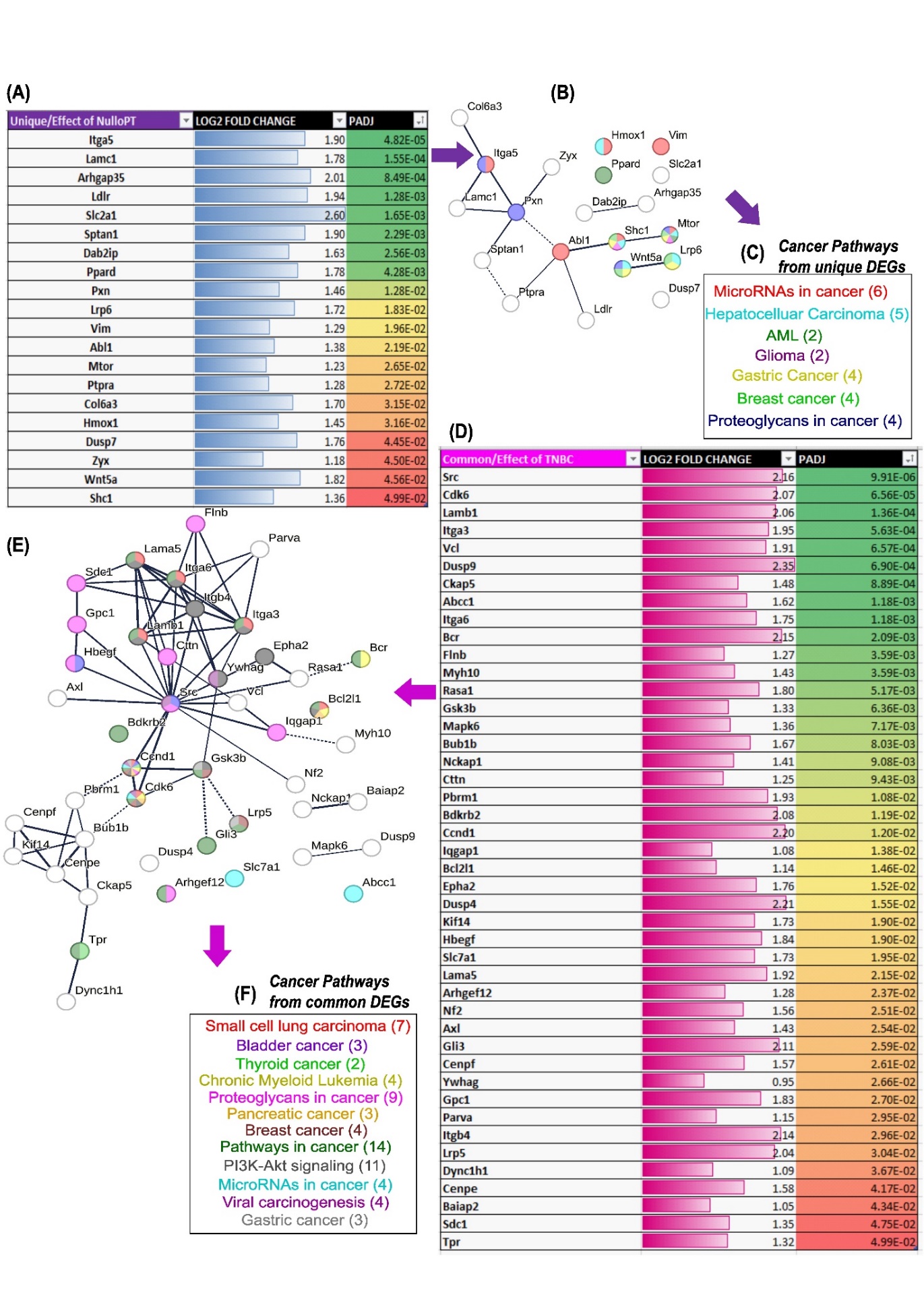


**Supplementary Fig 6:** Comparison of DEGs obtained from NulloPT treated TNBC tumors and untreated TNBC tumors (Fig. 7, group-a) vs, published DEGs comparing mouse 4T1 TNBC cells and mouse mammary tissue (Fig. 7, group-b). Based on FDR, (A-C) functional enrichment analysis of upregulated genes in mouse TNBC but downregulated in NulloPT-treated tumors, (D-F) genes downregulated in mouse TNBC but upregulated in NulloPT-treated tumors. (A) Functional enrichment analysis of top 5 GO terms with description from all GO categories (in red), and (B) pathways (in red) showing the number of genes and FDR values, and (C) the PPI interactome with nodes representing the DEGs. The top 5 GO-terms (D) and pathways (E) from functional enrichment analysis of DEGs that were downregulated in mouse TNBC cells but upregulated in NulloPT-treated mouse TNBC tumors, and (F) The PPI interactome with nodes depicting the DEGs.


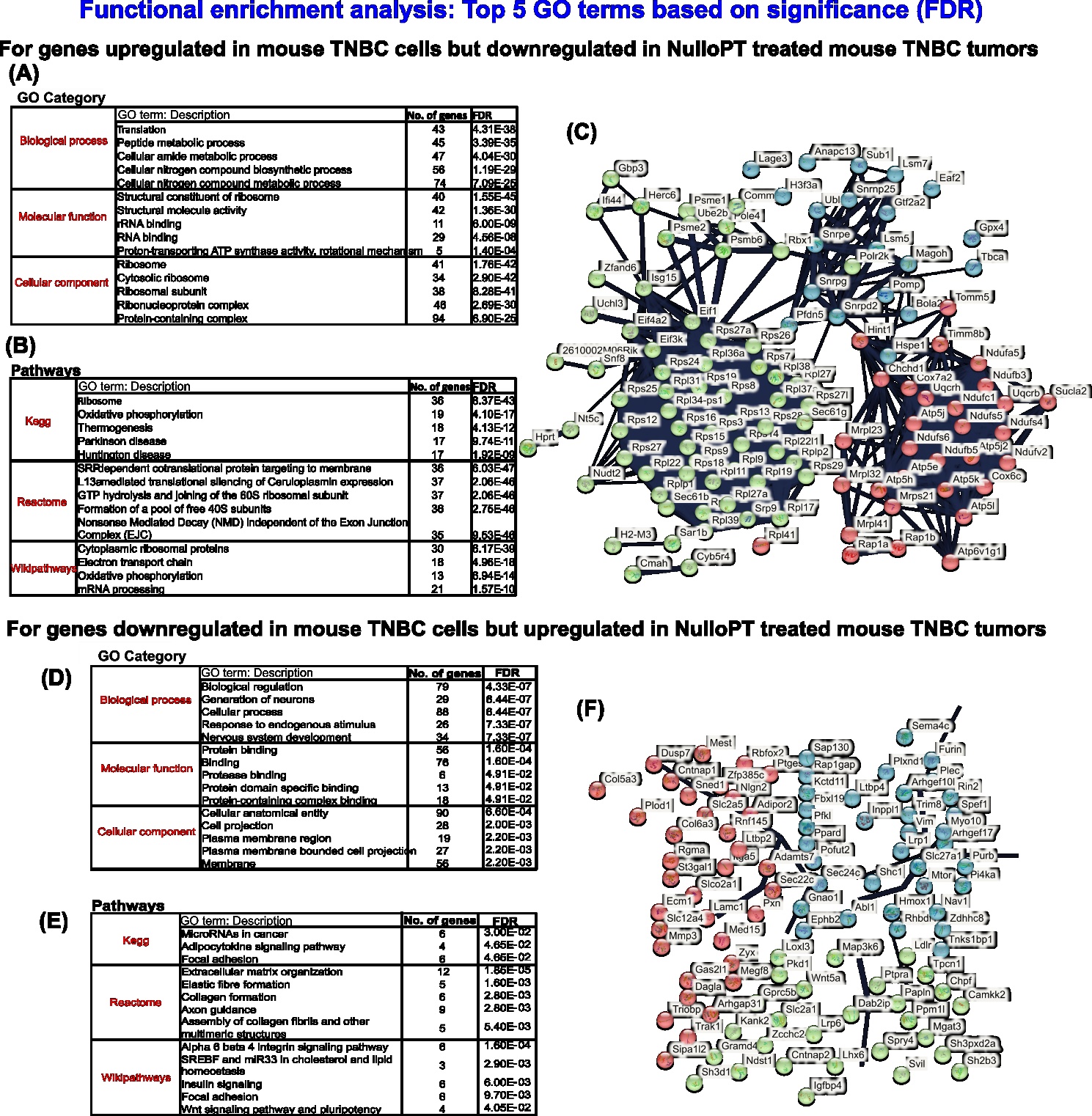


**Supplementary Table 1:**

| **DEGs** | **Log2 fold change** | **Function** | **Ref** |
| --- | --- | --- | --- |
| Adgrl1 | +3.4 | ADGRL1 (Adhesion G protein-coupled receptor L1), also known as LPHN1 (Latrophilin-1), encodes a member of the latrophilin subfamily of G-protein coupled receptors (GPCR). This cell surface receptor protein may function in cell adhesion, signal transduction and is involved in various biological processes, including neuronal development and synapse formation. Higher ADGRL1 expression is associated with poor prognosis in renal and pancreatic cancer and increased ADGRL1 expression has been linked to aggressive tumor characteristics in breast cancer. | [1-3] |
| Srp54b | +3.2 | SRP54B is a gene that encodes the Signal Recognition Particle 54kDa (SRP54) protein. SRP54 is an essential component of the signal recognition particle (SRP) complex, which plays a crucial role in protein targeting and trafficking within the cell. Point mutations in SRP54 have been recently shown to lead to a form of severe congenital neutropenia displaying symptoms overlapping with those of Shwachman-Diamond syndrome. The association of SRP54b and breast cancer has not been studied in great details. | [4, 5] |
| Arghgap22 | +3.0 | Arhgap22 gene encodes for a Rho GTPase Activating Protein 22, Or GRAF (GTPase regulator associated with focal adhesion kinase) This gene encodes a member of the GTPase activating protein family which activates a GTPase belonging to the RAS superfamily of small GTP-binding proteins. ARHGAP22 is an insulin-responsive protein and is reported to act as a tumor suppressor in breast cancer. It has been found to negatively regulate important processes in cancer progression. A low expression of ARHGAP22 has been correlated with poorer prognosis and reduced overall survival in certain subtypes of breast cancer. | [6, 7] |
| Prss22 | +2.9 | Prss22 (Protease, serine 22) is a gene that encodes a member of the trypsin family of serine protease enzyme. Previous reports show a decreased PRSS22 expression in TNBC whereas in hepatocellular carcinoma it is reported to be highly expressed. | [8-10] |
| Coro2a | +2.8 | Coro2A (Coronin 2A) is a gene that encodes a member of the WD repeat protein family. Members of this family are involved in a variety of cellular processes, including cell cycle progression, signal transduction, apoptosis, and gene regulation. This protein contains 5 WD repeats, and has a structural similarity with actin-binding proteins. It is involved in cytoskeletal dynamics and cellular processes such as cell migration, adhesion, and immune response. | [11, 12] |
| Igkv 3-4 | -11.8 | Igkv (Immunoglobulin kappa variable) is part of the immunoglobulin gene family involved in the production of antibodies. IGKV3-4 refers to a specific gene segment within the IGKV gene family. Alterations in the expression and function of IGKV, have been observed in various cancers, including breast cancer. | [13, 14] |
| Spib | -11.2 | SPIB (Spi-B Transcription Factor) protein belongs to the Ets family, it is a transcriptional activator that binds to the PU-box (5'-GAGGAA-3') and acts as a lymphoid-specific enhancer. Dysregulated SPIB activity may contribute to an altered tumor microenvironment and influence immune cell interactions. It has been observed that SPIB expression levels are higher in estrogen receptor-negative (ER-) breast tumors including TNBC. In breast cancer, SPIB has been implicated in promoting the production of pro-inflammatory cytokines and chemokines. Higher SPIB expression levels are correlated with poorer prognosis and reduced overall survival in breast cancer patients. | [15, 16] |
| Lax1 | -10.1 | Lax1 (Lymphocyte Transmembrane Adaptor 1) enables SH2 domain binding activity and protein kinase binding activity. It is involved in B cell activation, negative regulation of MAP kinase activity and negative regulation of T cell activation. Negatively regulates TCR (T-cell antigen receptor)-mediated signaling in T-cells and BCR (B-cell antigen receptor)-mediated signaling in B-cells. | [17] |
| Cd19 | -9.8 | CD19 (Cluster of differentiation 19) encodes a member of the immunoglobulin gene superfamily. Expression of this cell surface protein is restricted to B cell lymphocytes. This protein is a reliable marker for pre-B cells but its expression diminishes during terminal B cell differentiation and in antibody secreting plasma cells. | [18] |
| Myl3 | -9.7 | Myl3 (Myosin light chain 3) is a part of Myosin family of motor proteins that share the common features of ATP hydrolysis (ATPase enzyme activity), actin binding and potential for kinetic energy transduction. Although originally isolated from muscle cells, almost all eukaryotic cells are known to contain Myl3. | [19] |

**Supplementary Table 1**- List of top ten Upregulated (blue) and Downregulated (orange) DEGs and their function with references

**References:**

1. Lei P, Wang H, Yu L, Xu C, Sun H, Lyu Y, Li L, Zhang DL: **A correlation study of adhesion G protein-coupled receptors as potential therapeutic targets in Uterine Corpus Endometrial cancer**. Int Immunopharmacol 2022, **108**:108743.

2. White GR, Varley JM, Heighway J: **Isolation and characterization of a human homologue of the latrophilin gene from a region of 1p31.1 implicated in breast cancer**. Oncogene 1998, **17**(26):3513-3519.

3. Yasinska IM, Sakhnevych SS, Pavlova L, Teo Hansen Selno A, Teuscher Abeleira AM, Benlaouer O, Goncalves Silva I, Mosimann M, Varani L, Bardelli M et al: **The Tim-3-Galectin-9 Pathway and Its Regulatory Mechanisms in Human Breast Cancer**. Front Immunol 2019, **10**:1594.

4. Fan EM, Vagher J, Meznarich JA, Ubico EM, Goteti S, Peterson D, Rayes A, Maese LD: **Severe congenital neutropenia, SRP54 pathogenicity, and a framework for surveillance**. Am J Med Genet A 2023, **191**(5):1434-1441.

5. Li Y, Yang X, Zhao Z, Du J: **SRP54 mediates circadian rhythm-related, temperature-dependent gene expression in Drosophila**. Genomics 2022, **114**(6):110512.

6. Longatti A, Ponzoni L, Moretto E, Giansante G, Lattuada N, Colombo MN, Francolini M, Sala M, Murru L, Passafaro M: **Arhgap22 Disruption Leads to RAC1 Hyperactivity Affecting Hippocampal Glutamatergic Synapses and Cognition in Mice**. Mol Neurobiol 2021, **58**(12):6092-6110.

7. Mori M, Saito K, Sekine A, Hasebe R, Ohta Y: **Endosomal Localization of RacGAP Protein ARHGAP22 Regulates its GAP Activity in Human Melanoma Cells**. Anticancer Res 2022, **42**(12):5763-5771.

8. Solmi R, Ugolini G, Rosati G, Zanotti S, Lauriola M, Montroni I, del Governatore M, Caira A, Taffurelli M, Santini D et al: **Microarray-based identification and RT-PCR test screening for epithelial-specific mRNAs in peripheral blood of patients with colon cancer**. BMC Cancer 2006, **6**:250.

9. Song L, Li H, Ma RR, Liu S, Zhang GH, Guo XY, Zhao RN, Wu XJ, Zhang K, Gao P: **E2F1-initiated transcription of PRSS22 promotes breast cancer metastasis by cleaving ANXA1 and activating FPR2/ERK signaling pathway**. Cell Death Dis 2022, **13**(11):982.

10. McQuerry JA, Jenkins DF, Yost SE, Zhang Y, Schmolze D, Johnson WE, Yuan Y, Bild AH: **Pathway activity profiling of growth factor receptor network and stemness pathways differentiates metaplastic breast cancer histological subtypes**. BMC Cancer 2019, **19**(1):881.

11. Deng JL, Zhang HB, Zeng Y, Xu YH, Huang Y, Wang G: **Effects of CORO2A on Cell Migration and Proliferation and Its Potential Regulatory Network in Breast Cancer**. Front Oncol 2020, **10**:916.

12. Kase-Kato I, Asai S, Minemura C, Tsuneizumi K, Oshima S, Koma A, Kasamatsu A, Hanazawa T, Uzawa K, Seki N: **Molecular Pathogenesis of the Coronin Family: CORO2A Facilitates Migration and Invasion Abilities in Oral Squamous Cell Carcinoma**. Int J Mol Sci 2021, **22**(23).

13. Martorelli D, Guidoboni M, De Re V, Muraro E, Turrini R, Merlo A, Pasini E, Caggiari L, Romagnoli L, Spina M et al: **IGKV3 proteins as candidate "off-the-shelf" vaccines for kappa-light chain-restricted B-cell non-Hodgkin lymphomas**. Clin Cancer Res 2012, **18**(15):4080-4091.

14. Wang C, Xia M, Sun X, He Z, Hu F, Chen L, Bueso-Ramos CE, Qiu X, Yin CC: **IGK with conserved IGKappaV/IGKappaJ repertoire is expressed in acute myeloid leukemia and promotes leukemic cell migration**. Oncotarget 2015, **6**(36):39062-39072.

15. Huang Q, Liu J, Wu S, Zhang X, Xiao Z, Liu Z, Du W: **Spi-B Promotes the Recruitment of Tumor-Associated Macrophages via Enhancing CCL4 Expression in Lung Cancer**. Front Oncol 2021, **11**:659131.

16. Zhang H, Wang G, Zhou R, Li X, Sun Y, Li Y, Du W, Yan X, Yang J, Chang X et al: **SPIB promotes anoikis resistance via elevated autolysosomal process in lung cancer cells**. FEBS J 2020, **287**(21):4696-4709.

17. Genecards: **LAX1 Gene - Lymphocyte Transmembrane Adaptor 1**. In.

18. Genecards: **CD19 Gene - CD19 Molecule**.

19. Genecards: **MYL3 Gene - Myosin Light Chain 3**.
